# Impact of clinical pharmacist-led antimicrobial stewardship on antibiotic appropriateness, clinical outcomes, and antimicrobial consumption in hospital-acquired and ventilator-associated pneumonia: a randomized controlled trial

**DOI:** 10.3389/fpubh.2026.1700328

**Published:** 2026-04-07

**Authors:** Nawal Alsubaie, Muhammad Ilyas, Zafar Ali Shah, Ijaz Habib, Ammena Y. Binsaleh, Amani S. Alrossies

**Affiliations:** 1 Department of Pharmacy Practice, College of Pharmacy, Princess Nourah Bint Abdulrahman University, Riyadh, Saudi Arabia; 2 Department of Agricultural Chemistry & Biochemistry, The University of Agriculture, Peshawar, Pakistan; 3 Institute of Public Health and Social Sciences, Khyber Medical University, Peshawar, Pakistan; 4 United Nations World Food Programme, Peshawar, Pakistan

**Keywords:** antimicrobial resistance, antimicrobial stewardship, appropriate prescribing, clinical pharmacist, defined daily doses, healthcare-associated pneumonia, hospital-acquired pneumonia, ventilator-associated pneumonia

## Abstract

**Background:**

Hospital-acquired pneumonia (HAP) and ventilator-associated pneumonia (VAP) are major drivers of inappropriate antimicrobial use and resistance in hospitals. Clinical pharmacist-led antimicrobial stewardship programs (ASPs) are promising interventions for optimizing antimicrobial prescriptions and combating antimicrobial resistance.

**Methods:**

This prospective randomized controlled trial was conducted over 18 months (from April 2023 to October 2024) at a tertiary care hospital. Adult patients with HAP or VAP were randomized to receive either pharmacist-led ASP interventions (*n* = 366) or standard care (*n* = 334) in a clinical trial setting. The intervention comprised daily medication reviews, culture-guided optimization, therapeutic drug monitoring, duration optimization, adverse event monitoring, and multidisciplinary communication training. The primary outcome was an appropriate antimicrobial prescription on day three.

**Results:**

The intervention group demonstrated significantly higher rates of appropriate antimicrobial prescriptions (77.6% vs. 60.8%; odds ratio [OR] = 2.26, 95% confidence interval [CI]: 1.65–3.09; *p* < 0.001). Clinical cure rates were higher in the intervention group (84.4% vs. 74.3%; OR = 1.86, 95% CI: 1.27–2.72; *p* = 0.001). The mean hospital length of stay was reduced by 2.83 days (14.64 ± 5.48 vs. 17.47 ± 7.22 days; *p* < 0.001). Total antimicrobial treatment duration decreased by 10.9% (11.98 ± 5.80 vs. 13.45 ± 6.12 days; *p* < 0.001). Antimicrobial-related adverse events occurred less frequently in the intervention group (8.7% vs. 14.1%; *p* = 0.04).

**Conclusion:**

Clinical pharmacist-led antimicrobial stewardship programs (ASPs) significantly reduced antimicrobial exposure and healthcare utilization, improving the appropriateness of antimicrobial prescriptions and clinical outcomes. These findings highlight the importance of ASPs as an evidence-based strategy to address antimicrobial resistance and optimize patient care in HAP.

## Graphical abstract

**Figure:**
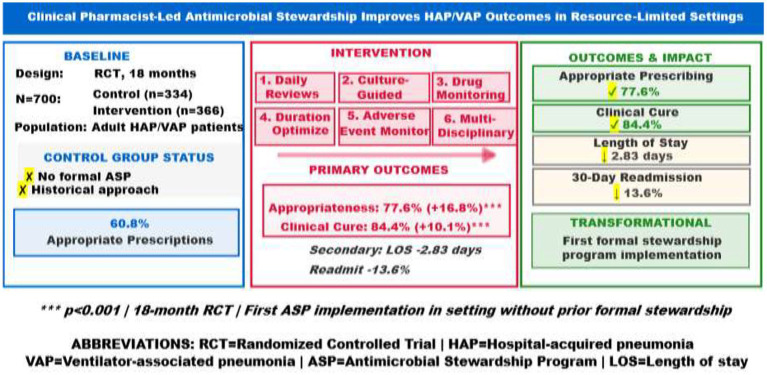
This graphical abstract summarizes the findings of a randomized controlled trial demonstrating that clinical pharmacist-led antimicrobial stewardship significantly improves the appropriateness of antimicrobial prescriptions and clinical cure rates, and reduces hospital length of stay in patients with hospital-acquired pneumonia HAP and ventilator-associated pneumonia (VAP) in a resource-limited healthcare setting without a prior formal stewardship infrastructure.

## 1 Introduction

Healthcare-associated infections, such as healthcare-associated pneumonia, hospital-acquired pneumonia (HAP), and ventilator-associated pneumonia (VAP), pose critical global health challenges (1). These infections create a significant clinical and economic burden through prolonged hospitalization, increased mortality, and healthcare costs (2, 3). The complexity of managing HAP and VAP lies in the need for realistic, broad-spectrum antimicrobial control while reducing selection pressure for multidrug-resistant organisms, particularly extended-spectrum *β*-lactamase (ESBL)-producing *Enterobacteriaceae*, carbapenem-resistant pathogens, and methicillin-resistant *Staphylococcus aureus* (MRSA) (4, 5).

Current evidence indicates that 40–60% of antimicrobial prescriptions in hospitalized patients with pneumonia may be clinically inappropriate, characterized by incorrect drug selection, suboptimal dosing, excessive duration, or failure to de-escalate therapy based on microbiological results (6). This inappropriate use directly contributes to the development of antimicrobial resistance, healthcare-associated infections, including *Clostridioides difficile* infection, and adverse drug events. The World Health Organization has identified antimicrobial resistance as one of the top 10 global public health threats, with pneumonia pathogens being among the priority pathogens requiring urgent attention (7).

Antimicrobial stewardship programs (ASPs) have emerged as evidence-based interventions to optimize antimicrobial use, improve patient outcomes, and preserve efficacy (8). The Centers for Disease Control and Prevention and the Infectious Diseases Society of America recommend implementing comprehensive stewardship programs that incorporate prospective audits and feedback, formulary restrictions, clinical guidelines, and multidisciplinary collaboration (9). Clinical pharmacists possess specialized expertise in antimicrobial pharmacokinetics (PK), pharmacodynamics (PD), and resistance mechanisms, positioning them ideally to lead stewardship initiatives (10).

Systematic reviews and meta-analyses have demonstrated that pharmacist-led ASPs reduce antimicrobial consumption by 8–15%, decrease hospital length of stay by 1.0–2.5 days, and improve clinical outcomes without increasing mortality (10–12). However, robust evidence from randomized controlled trials specifically evaluating pharmacist-led interventions for healthcare-associated pneumonia remains limited, particularly regarding resistance prevention and antimicrobial consumption. Furthermore, the majority of the existing studies originate from high-resource settings with well-established antimicrobial stewardship infrastructures, potentially limiting their generalizability to diverse healthcare systems.

Despite the growing evidence supporting antimicrobial stewardship, several critical knowledge gaps persist. First, the majority of the existing studies are observational or quasi-experimental, with few randomized controlled trials specifically evaluating pharmacist-led interventions for healthcare-associated pneumonia. Second, prior studies often report aggregate outcomes without detailing which specific pharmacist interventions (e.g., de-escalation, dose optimization, therapeutic drug monitoring) contribute the majority to the observed benefits. Third, limited data are available from middle-income countries with healthcare infrastructures and antimicrobial resistance patterns different from those in high-income settings.

This randomized controlled trial evaluated the effectiveness of a comprehensive pharmacist-led antimicrobial stewardship intervention in patients with HAP and VAP. This study assessed the appropriateness of antimicrobial prescribing, clinical outcomes, antimicrobial consumption, and healthcare utilization in a middle-income healthcare setting to inform the implementation of future evidence-based stewardship programs.

## 2 Materials and methods

### 2.1 Study design and setting

This prospective, randomized controlled trial was conducted over 18 months (from April 2023 to October 2024) in the internal medicine, pulmonology, and intensive care departments of Khyber Teaching Hospital, Peshawar, Pakistan, a 400-bed tertiary care academic medical center serving as a referral center for the Khyber Pakhtunkhwa province (population: ~35 million). The hospital provides quaternary care, including solid organ transplantation, advanced cardiac care, and neurosurgery.

Pakistan is a middle-income country with high antimicrobial resistance rates (ESBL-producing Enterobacteriaceae prevalence >60% and carbapenem resistance >30% in our center) and limited antimicrobial stewardship infrastructure.

#### 2.1.1 Institutional antimicrobial stewardship baseline

At the commencement of the study in April 2023, the Khyber Teaching Hospital lacked a formalized antimicrobial stewardship program. Prior to this investigation, the hospital lacked dedicated clinical pharmacy personnel focused on antimicrobial stewardship, a systematic daily review of antimicrobial prescriptions, and formal antimicrobial prescribing guidelines. However, informal institutional practices, therapeutic drug monitoring services for antimicrobials, and formal audit and feedback mechanisms were in place.

A baseline audit conducted from January to March 2023 indicated that adherence to institutional guidelines when followed informally was only 58%. Institutional antimicrobial guidelines were formally updated in December 2022 based on local antibiogram data, the Infectious Diseases Society of America (IDSA)/American Thoracic Society (ATS) 2016 guidelines, and local resistance patterns. These guidelines were disseminated to all physicians via the hospital intranet, educational sessions, and pocket-reference cards. However, these guidelines were not systematically enforced or monitored using formal mechanisms. The control group received standard care with access to updated guidelines but without a systematic pharmacist review or intervention. Conversely, the intervention group benefited from additional clinical pharmacist-led stewardship, marking the initial implementation of a formal, structured antimicrobial stewardship program at this hospital.

#### 2.1.2 Institutional antimicrobial guidelines for HAP/VAP management

The revised institutional guidelines for the management of HAP/VAP were formulated based on local antibiogram data from the Khyber Teaching Hospital Pathology/Microbiology Department (2021–2022), the IDSA/ATS 2016 Clinical Practice Guidelines for HAP/VAP management, and local resistance prevalence patterns (8, 13, 14).

##### 2.1.2.1 Empirical therapy recommendations (based on local antibiogram)

For non-severe hospital-acquired pneumonia (HAP) without risk factors for multidrug-resistant (MDR) organisms, the recommended treatment includes ampicillin-sulbactam or ceftriaxone. In cases of severe HAP or ventilator-associated pneumonia (VAP) with MDR risk factors, the suggested regimen is piperacillin-tazobactam or carbapenems (imipenem or meropenem) in combination with vancomycin or ceftazidime. For cultures positive for *Pseudomonas aeruginosa,* an anti-pseudomonal *β*-lactam (piperacillin-tazobactam or carbapenem) should be administered along with a fluoroquinolone or aminoglycoside. Vancomycin or linezolid is recommended for suspected or confirmed methicillin-resistant *Staphylococcus aureus* (MRSA) infections. Carbapenem monotherapy is recommended for infections caused by extended-spectrum *β*-lactamase (ESBL)-producing *Enterobacteriaceae*.

##### 2.1.2.2 De-escalation principles

Upon obtaining culture and susceptibility results, typically within 48–72 h, the following actions should be taken. Transition from broad-spectrum to narrow-spectrum antimicrobial agents that are appropriate for the identified organism and its susceptibilities; discontinue vancomycin if Methicillin-resistant *Staphylococcus aureus* (MRSA) is not isolated and *β*-lactam antibiotics demonstrate susceptibility; shift from dual therapy to monotherapy when monotherapy offers adequate coverage; cease the use of redundant antimicrobial agents that target the same organism; and eliminate unnecessary agents from combination therapy.

##### 2.1.2.3 Duration recommendations

The recommended duration of therapy for hospital-acquired pneumonia (HAP) is 7–8 days, whereas for ventilator-associated pneumonia (VAP), it ranges from 7 to 14 days, contingent upon the specific pathogen and the patient’s clinical response. These guidelines are consistent with the hospital’s Infection Prevention and Control Manual (*Manual for Hospital Infection Prevention and Control, Revised July 2020*, Section 14: Antibiotic Policy) and are followed by all institutional stakeholders.

### 2.2 Participants and eligibility criteria

Adult patients (≥18 years of age) diagnosed with HAP or VAP were eligible for inclusion. HAP was defined as pneumonia occurring (≥48 h) after hospital admission, characterized by new pulmonary infiltrates on chest imaging plus ≥2 of the following: fever (≥38.3 °C), leukocytosis (≥12,000 cells/μL), leukopenia (≤4,000 cells/μL), or purulent respiratory secretions. VAP was defined as pneumonia occurring (≥48 h) after endotracheal intubation and mechanical ventilation (13).

Additional inclusion criteria were prescription of antimicrobial therapy for pneumonia, an expected hospital stay of ≥5 days, and availability for follow-up. The exclusion criteria included severe immunocompromising conditions (HIV with CD4 + count <200 cells/μL, active malignancy receiving chemotherapy, and solid organ transplant recipients), pregnancy, enrollment in other clinical trials, palliative care status, and documented allergies to multiple antimicrobial classes that limited therapeutic options.

### 2.3 Randomization and allocation concealment

#### 2.3.1 Randomization and blinding

Participants were randomized in a 1:1 ratio using computer-generated block randomization with variable block sizes (4, 6, and 8) to ensure balanced allocation while preventing the prediction of upcoming assignments. Randomization was stratified by baseline Acute Physiology and Chronic Health Evaluation II (APACHE II) score (≤10 vs. >10) to balance disease severity between the groups.

The randomization sequence was generated prior to the study commencement using R software (version 4.2.2) and was concealed in sequentially numbered, sealed, opaque envelopes. An independent research coordinator, not involved in patient care, held these envelopes. Upon enrollment, the coordinator opened the next sequential envelope to reveal the group assignments. Treating physicians, outcome assessors, and laboratory staff remained blinded to the block sizes and allocation sequence throughout the study to ensure allocation concealment and prevent selection bias.

### 2.4 Interventions

#### 2.4.1 Control group (*n* = 334)

Control patients received standard medical care and antimicrobial prescriptions from attending physicians in accordance with the institutional guidelines. All physicians had access to updated institutional antimicrobial guidelines through educational sessions (conducted in January 2023), pocket reference cards, and the hospital’s electronic systems. Standard care included empirical antimicrobial selection based on local antibiograms, patient-specific risk factors, and clinical presentation, with modifications at the physician’s discretion based on culture results and clinical response.

#### 2.4.2 Intervention group (*n* = 366)

Patients received comprehensive clinical pharmacist-led ASP interventions in addition to standard care.

#### 2.4.3 Pharmacist training

All participating clinical pharmacists completed structured training in antimicrobial pharmacology, resistance mechanisms, diagnostic stewardship, pharmacokinetics/pharmacodynamics optimization, and communication skills. Pharmacists had postgraduate clinical pharmacy training and a minimum of 2 years of clinical experience.

#### 2.4.4 Daily medication review

Qualified clinical pharmacists conducted comprehensive daily medication reviews, assessing the appropriateness of antimicrobials, dosing optimization based on patient-specific factors (renal function, hepatic status, and body weight), drug interaction screening, and therapy duration.

#### 2.4.5 Culture-guided optimization

Pharmacists provided evidence-based recommendations for antimicrobial selection, de-escalation, and discontinuation based on the culture and sensitivity results, local antibiograms, and patient-specific factors. Emphasis was placed on narrowing the spectrum when possible and discontinuing redundant antimicrobial therapy.

#### 2.4.6 Therapeutic drug monitoring

When applicable, pharmacists monitor serum drug levels for antimicrobials requiring therapeutic drug monitoring (e.g., vancomycin and aminoglycosides) and adjust dosing regimens to optimize efficacy while minimizing toxicity.

#### 2.4.7 Duration optimization

Pharmacists recommend the optimal treatment duration based on clinical response, microbiological data, and evidence-based guidelines, emphasizing the shortest effective duration to minimize the development of resistance and drug-related adverse effects.

#### 2.4.8 Adverse event monitoring

Systematic monitoring of antimicrobial-related adverse events, including nephrotoxicity (≥50% increase in serum creatinine), hepatotoxicity (≥3-fold increase in liver enzymes), hematological effects, and drug–drug interactions, with prompt intervention when identified.

#### 2.4.9 Multidisciplinary communication

Daily consultation with medical teams through structured communication formats, written recommendations in medical records, and participation in multidisciplinary rounds when feasible.

### 2.5 Outcome measures

#### 2.5.1 Primary outcome

Appropriate antimicrobial prescription on day 3 of therapy was assessed retrospectively by two independent infectious disease specialists who were blinded to the group allocation. A prescription was classified as “Appropriate” if it met all the following five criteria, according to the hospital’s institutional guidelines for HAP/VAP management (updated December 2022, based on local resistance patterns, IDSA/ATS 2016 guidelines, and institutional antibiogram) (8).

##### 2.5.1.1 Criterion 1 (dug selection)

The selected antimicrobial agents provided adequate coverage of the cultured organisms, as determined by culture and susceptibility results. Alternatively, if cultures are not yet available or yield negative results, the selection provides appropriate empirical coverage in accordance with institutional guidelines, considering the patient’s risk category (non-severe HAP vs. severe HAP/VAP with MDR risk factors). The selection also considers local resistance patterns, such as the high prevalence of ESBL-producing and carbapenem-resistant organisms in the community. Furthermore, adherence to the institutional MRSA control policy is maintained, including the use of vancomycin or linezolid for MRSA and the avoidance of *β*-lactam antibiotics in MRSA cases. Compliance with the institutional policy for controlling multidrug-resistant gram-negative (MDRGN) organisms was also observed.

##### 2.5.1.2 Criterion 2 (dosing)

Dosage was appropriately adjusted based on patient-specific pharmacokinetic factors, including renal function (assessed by creatinine clearance and the Kidney Disease: Improving Global Outcomes [KDIGO] classification of kidney disease), hepatic function (evaluated using the Child-Pugh score, if applicable), body weight, age, and comorbidities. The dosing regimen adheres to pharmacokinetic/pharmacodynamic (PK/PD) principles relevant to the specific site of infection and organism involved, such as time vs. concentration dependent killing. Furthermore, the dosage was aligned with the guidelines and recommendations of institutional pharmacy services.

##### 2.5.1.3 Criterion 3 (route of administration)

The route of administration, whether intravenous or oral, should be selected based on the severity of the infection and the patient’s clinical status. A documented plan for conversion from intravenous to oral administration (IV=PO) should be established when clinically appropriate. The rationale for selecting the administration route must be documented in the clinical notes.

##### 2.5.1.4 Criterion 4 (planned duration)

The prescribed duration of antimicrobial therapy was consistent with evidence-based guidelines. For hospital-acquired pneumonia (HAP), the intended duration is 7–8 days of appropriate treatment, whereas for ventilator-associated pneumonia (VAP), the duration ranges from 7 to 14 days, depending on the type of pathogen and the clinical response. The duration was adjusted as necessary based on clinical response and culture/susceptibility results. The rationale for selecting the duration is documented in the clinical notes.

##### 2.5.1.5 Criterion 5 (de-escalation and combination therapy appropriateness)

In patients administered multiple antimicrobial agents, dual or combination therapy is warranted under specific circumstances. These circumstances include either a confirmed polymicrobial infection necessitating synergistic coverage or a documented clinical indication for combination therapy, such as a suspected severe infection or an immunocompromised patient requiring broad-spectrum coverage. In instances where de-escalation is feasible, meaning a narrower-spectrum agent adequately covers the identified organism, the prescriber should have a documented plan for de-escalation upon receipt of culture results, or de-escalation should already have been implemented. Furthermore, unnecessary duplication of coverage should be avoided, such as the use of two agents targeting the same organism without specific clinical justification.

##### 2.5.1.6 Scoring system (all-or-nothing approach)

A prescription was deemed “Appropriate” only when all five criteria were satisfied. Conversely, if any single criterion was unmet, the prescription was classified as “Inappropriate” irrespective of its performance on other criteria. This binary classification mirrors the clinical reality that a prescription with appropriate drug selection but suboptimal dosing remains clinically inappropriate because of the potential for therapeutic failure due to inadequate drug levels. Similarly, a prescription with the correct drug choice but excessive duration is clinically inappropriate because of unnecessary exposure, increased resistance, and adverse effects.

Furthermore, prescribing suitable empirical therapy that was not de-escalated despite culture results indicating a narrower option is also clinically inappropriate. This stringent all-or-nothing approach aligns with stewardship principles that necessitate optimization across all domains rather than mere adequacy.

##### 2.5.1.7 Assessment methodology

A retrospective assessment of appropriateness was conducted on the third day of antimicrobial therapy. This specific time point was chosen because preliminary microbiological results, such as Gram staining and rapid diagnostics, are generally available within 24–48 h, whereas complete culture and susceptibility results are typically finalized within 48–72 h. This juncture is a critical decision point, as recommended by international guidelines, for the re-evaluation of empirical therapy. It also facilitates the assessment of early opportunities for de-escalation, optimization of dosing, and appropriateness of therapy duration.

The assessment was performed by two independent infectious disease specialists from the Department of Internal Medicine/Infectious Diseases, both of whom were blinded to the group allocation and study hypothesis. Each specialist independently reviewed all prescription data, culture results, patient-specific factors, and clinical contexts to determine their appropriateness.

##### 2.5.1.8 Inter-rater agreement

Cohen’s kappa coefficient for inter-rater agreement was calculated as *κ* = 0.89 (*p* < 0.001), signifying excellent concordance between assessors. In cases of disagreement (11 cases, 1.6%), discrepancies were resolved through consultation with a third infectious disease expert who independently applied the same criteria. The final classification was determined by consensus among the three consultants.

##### 2.5.1.9 Institutional basis

The five criteria are directly aligned with the hospital’s Infection Prevention and Control Manual (*Manual for Hospital Infection Prevention and Control, Revised July 2020*, Section 14: Antibiotic Policy), which stipulates that antimicrobial stewardship should encompass a systematic review of the following: (1) appropriate drug selection, (2) optimal dosing, (3) appropriate route of administration, (4) adequate treatment duration, and (5) appropriate de-escalation and combination therapies.

#### 2.5.2 Secondary outcomes

##### 2.5.2.1 Clinical cure rate

Complete resolution of pneumonia symptoms and signs, including temperature normalization (<38 °C for ≥24 h), leukocyte count improvement, resolution of purulent secretions, and chest imaging improvement within 14 days of treatment initiation without the need for alternative antimicrobial therapy.

##### 2.5.2.2 Length of hospital stay

Total days from pneumonia diagnosis to hospital discharge or death.

##### 2.5.2.3 Total antimicrobial treatment days

Cumulative duration of all systemic antimicrobial therapies for the pneumonia episode, including empirical therapy and subsequent modifications.

##### 2.5.2.4 Antimicrobial consumption

Defined daily doses (DDDs) per 100 patient-days according to the World Health Organization (WHO) Anatomical Therapeutic Chemical Classification and Defined Daily Dose (ATC/DDD) methodology (12).

##### 2.5.2.5 30-day hospital readmission

Unplanned readmission to any hospital within 30 days of initial discharge for any reason.

##### 2.5.2.6 Antimicrobial-related adverse events

These include nephrotoxicity, hepatotoxicity, hematological toxicity, *C. difficile* infection, and drug–drug interactions requiring intervention.

##### 2.5.2.7 Healthcare-associated infections

Secondary infections occurring during hospitalization, including central line-associated bloodstream infections and catheter-associated urinary tract infections.

### 2.6 Statistical analysis

The sample size was calculated based on the improvement in appropriate antimicrobial prescription, 60% (control) to 75% (intervention), with power (80%) and two-sided significance (*α* = 0.05). The target sample size was 700, based on a 10% attrition rate. Continuous variables were reported as mean ± standard deviation (SD) and compared using independent *t*-tests after confirming the data’s normal distribution. Categorical variables, such as frequency and percentage, were compared using the chi-square test.

Multivariate logistic regression analysis was performed to identify independent predictors of clinical cure on day 14, after adjusting for potential confounding variables. The model incorporated the following covariates: treatment group (pharmacist-led ASP intervention vs. standard care), age (continuous, per year), Charlson comorbidity score, pneumonia severity index (PSI), and pneumonia type (HAP vs. VAP). Including pneumonia type (HAP vs. VAP) as a covariate account for potential differences in disease severity, treatment response, and outcomes between these two distinct clinical entities, with VAP generally associated with higher severity and potentially lower clinical cure rates than HAP. This variable was included to ensure that the observed treatment benefits were consistent across both types of pneumonia. Variables with *p* < 0.10 in the univariate analysis were included in the multivariable models. Goodness-of-fit was evaluated using the Hosmer–Lemeshow test. A two-sided *p* < 0.05 was considered to be statistically significant.

## 3 Results

### 3.1 Patient characteristics and study flow

**Figure 1:**
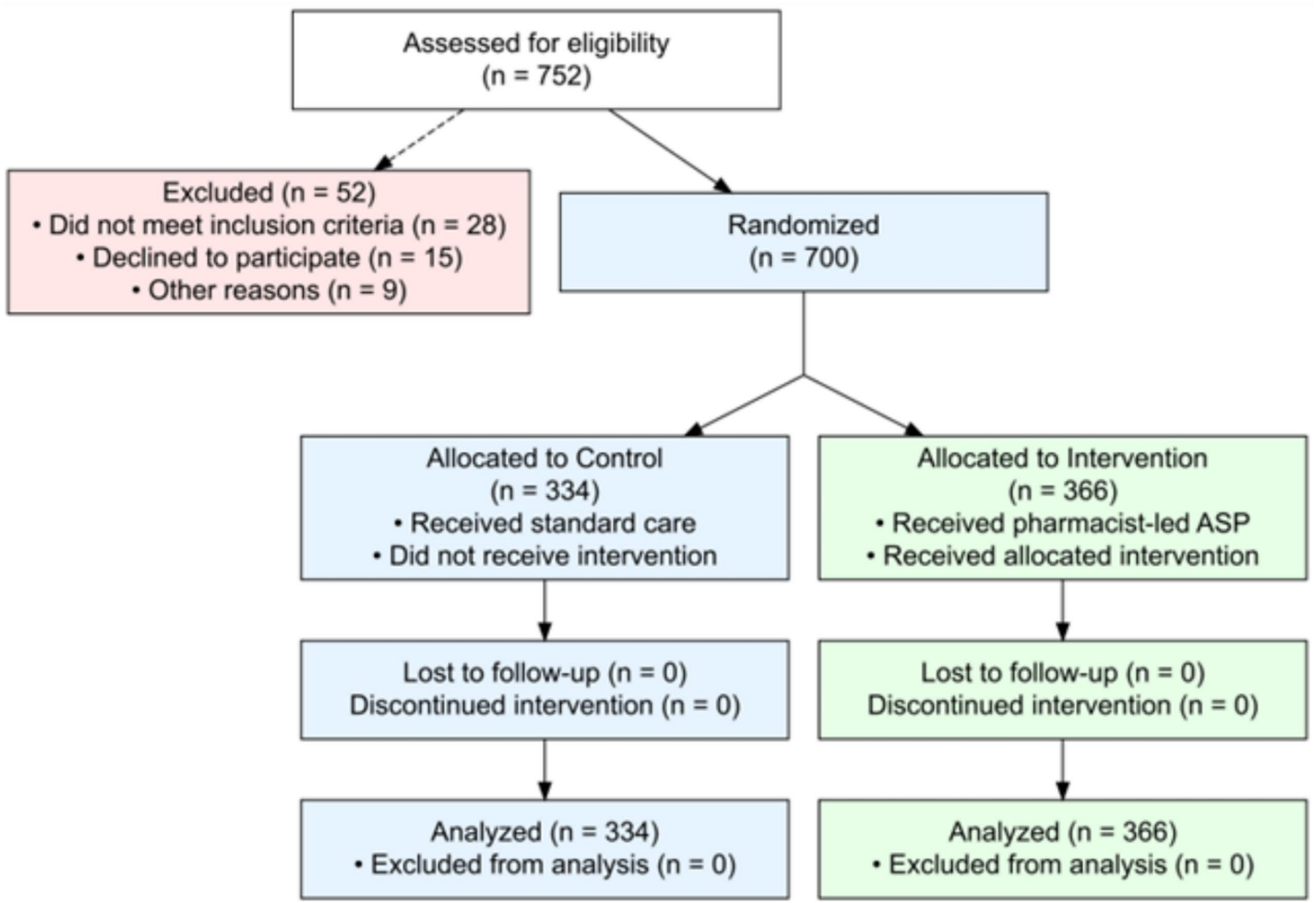
Flow diagram showing patient enrollment, randomization, and completion of the clinical pharmacist-led antibiotic stewardship trial for HAP and VAP.

A total of 700 patients were enrolled and randomized, with 334 and 366 patients in the control and intervention groups, respectively (14). All randomized patients completed the study, with no loss to follow-up for the primary endpoints (Figure 1). The baseline demographic and clinical characteristics were well-balanced between the groups (Tables 1, 2). The overall population had a mean age of 65.17 ± 11.96 years, with 54.8% of patients being male. The mean Charlson comorbidity index was 4.48 ± 2.91, indicating a moderate burden of comorbidities. The mean pneumonia severity index was 3.07 ± 1.43. HAP accounted for 59.1% (414 patients), whereas VAP accounted for 40.9% (286 patients).

**Table 1:** Baseline patient characteristics.

Characteristic	Control group (*n* = 334)	Intervention group (*n* = 366)	*p*-value
Demographics			
Age, years (mean ± SD)	65.2 ± 12.1	65.1 ± 11.8	0.891
Male gender, *n* (%)	189 (56.6)	198 (54.1)	0.482
Comorbidity scores			
Charlson Score (mean ± SD)	4.5 ± 2.9	4.5 ± 2.9	0.984
PSI Score (mean ± SD)	3.1 ± 1.4	3.0 ± 1.4	0.443
Type of pneumonia			
HAP, *n* (%)	198 (59.3)	216 (59.0)	0.939
VAP, *n* (%)	136 (40.7)	150 (41.0)	0.939

Data are presented as mean ± standard deviation or numbers (percentages).

**Table 2:** Baseline microbiological characteristics and culture results.

Characteristic	Control (*n* = 334)	Intervention (*n* = 366)	*p*-value
Culture status			
Cultures obtained, *n* (%)	312 (93.4)	339 (92.6)	0.685
Positive cultures, *n* (%)	267 (85.6)	287 (84.7)	0.741
Polymicrobial infection, *n* (%)	43 (16.1)	51 (17.8)	0.599
Organisms isolated (*n* = 554 total isolates)			
Gram-negative organisms			
*Klebsiella pneumoniae*, *n* (%)	78 (29.2)	89 (31.0)	0.634
*Pseudomonas aeruginosa*, *n* (%)	67 (25.1)	71 (24.7)	0.920
*Acinetobacter baumannii*, *n* (%)	45 (16.9)	42 (14.6)	0.465
*Escherichia coli*, *n* (%)	34 (12.7)	38 (13.2)	0.859
Other Gram-negative, *n* (%)	18 (6.7)	21 (7.3)	0.784
Gram-positive organisms			
MRSA, *n* (%)	15 (5.6)	18 (6.3)	0.742
MSSA, *n* (%)	10 (3.7)	8 (2.8)	0.530
Resistance patterns			
ESBL-producing *Enterobacteriaceae*, *n* (%)	89 (33.3)	98 (34.1)	0.833
Carbapenem-resistant organisms, *n* (%)	76 (28.5)	81 (28.2)	0.949
MDR *P. aeruginosa*, *n* (%)	43 (16.1)	39 (13.6)	0.398
MDR *A. baumannii*, *n* (%)	38 (14.2)	35 (12.2)	0.478
MRSA, *n* (%)	15 (5.6)	18 (6.3)	0.742

ESBL, extended-spectrum β-lactamase; MDR, multidrug resistant; MRSA, methicillin-resistant *Staphylococcus aureus*; MSSA, methicillin-sensitive *Staphylococcus aureus*. The organisms isolated represented the percentage of patients with positive cultures (control, *n*=267; intervention, *n*=287). The proportions of isolated organisms were calculated as the percentage of patients with documented isolated organisms. The total may exceed 100% because some patients had multiple organisms isolated from their samples. Statistical comparisons were performed using the chi-square or Fisher's exact test, where appropriate.

### 3.2 Primary outcome

#### 3.2.1 Appropriate antimicrobial prescribing

The intervention group demonstrated significantly higher rates of appropriate antimicrobial prescriptions on day 3 than the control group (Table 3). A total of 284 patients (77.6%) in the intervention group and 203 patients (60.8%) in the control group received appropriate antimicrobial therapy, representing a 16.8 percentage-point improvement (χ^2^ = 23.33, *p* < 0.001) (8).

**Table 3:** Primary and secondary clinical outcomes.

Outcome	Control (*n* = 334)	Intervention (*n* = 366)	Effect size	*p*-value
Primary outcome				
Appropriate prescribing (day 3), *n* (%)	203 (60.8%)	284 (77.6%)	OR = 2.26 (95% CI: 1.65–3.09)	<0.001
Secondary Outcomes				
Clinical cure (day 14), *n* (%)	248 (74.3%)	309 (84.4%)	OR = 1.86 (95% CI: 1.27–2.72)	0.001
Length of stay (days), mean ± SD	17.47 ± 7.22	14.64 ± 5.48	Mean difference = −2.83 (95% CI: −3.76 to −1.90)	<0.001
Total antimicrobial days, mean ± SD	13.89 ± 6.20	10.23 ± 4.78	Mean difference = −3.66 (95% CI: −4.48 to −2.84)	<0.001
30-day readmission, *n* (%)	71 (21.3%)	28 (7.7%)	OR = 0.30 (95% CI: 0.19–0.48)	<0.001
Adverse events, *n* (%)	47 (14.1%)	32 (8.7%)	OR = 0.58 (95% CI: 0.36–0.94)	0.026

χ2 = 23.33, df = 1, *p* < 0.001; OR = 2.26 (95% CI: 1.65–3.09). χ2 = 11.12, df = 1, *p* = 0.001; OR = 1.86 (95% confidence interval; [CI]: 1.27–2.72). Statistical analysis revealed a highly significant difference between the two groups (*p* < 0.001).

#### 3.2.2 Appropriateness domain breakdown

To identify the specific stewardship domains that contributed to improved appropriateness within the intervention group, we conducted a separate analysis of each criterion’s performance.

In the intervention group, among the 284 prescriptions deemed appropriate, the outcomes were as follows: drug selection was correct in 284 of 284 cases (100%), dosing was accurate in 282 of 284 cases (99.3%), route of administration was appropriate in 280 of 284 cases (98.6%), duration of treatment was suitable in 276 of 284 cases (97.2%), and de-escalation or combination therapy was correctly applied in 271 of 284 cases (95.4%).

In the control group, among the 203 appropriate prescriptions, the following observations were made. Drug selection was appropriate in 203 of 334 cases (60.8%), dosing was correct in 147 of 334 cases (44.0%), the route of administration was suitable in 168 of 334 cases (50.3%), the duration was appropriate in 156 of 334 cases (46.7%), and de-escalation or combination therapy was correctly applied in 134 of 334 cases (40.1%).

The most frequently deficient domains in the control group were:

##### 3.2.2.1 De-escalation/combination therapy

In the control group, 59.9% of the patients (200/334) did not meet this criterion. There was a failure to de-escalate to a narrower spectrum of antibiotics, despite culture results indicating susceptibility to more targeted agents. Piperacillin-tazobactam therapy was continued even after the cultures identified susceptibility to ceftriaxone.

##### 3.2.2.2 Duration inappropriateness

In the control group, 53.3% of patients (178/334) did not meet the specified criteria. This was attributed to the duration of antimicrobial therapy being either insufficient (less than 7 days with an inadequate response) or excessively prolonged beyond the guideline recommendations without a clinical justification.

##### 3.2.2.3 Institutional pharmacy guidelines

In the control group, 56.0% of patients (187/334) failed to meet this criterion due to inadequate dose adjustments based on renal function, body weight, or pharmacokinetic/pharmacodynamic (PK/PD) principles.

##### 3.2.2.4 Route selection issues

In the control group, 49.7% of patients (166/334) did not meet this criterion because of either a delayed transition from intravenous to oral administration or the selection of an inappropriate route given the severity of the disease. These specific deficiencies were directly targeted by the pharmacist-led intervention through a systematic review of culture results at 48–72 h to identify de-escalation opportunities. Duration assessment and notification to the prescriber if therapy extends beyond guideline recommendations.

Therapeutic drug monitoring is employed to optimize dosing based on patient-specific factors, such as renal function. Intravenous-to-oral conversion was facilitated when clinically appropriate. The intervention group exhibited significant improvements across all five domains, with over 95% of the participants meeting each criterion. This comprehensive enhancement across all domains indicates that pharmacist-led stewardship effectively addresses multiple deficiency patterns simultaneously rather than merely improving selected aspects of prescribing.

**Figure 2:**
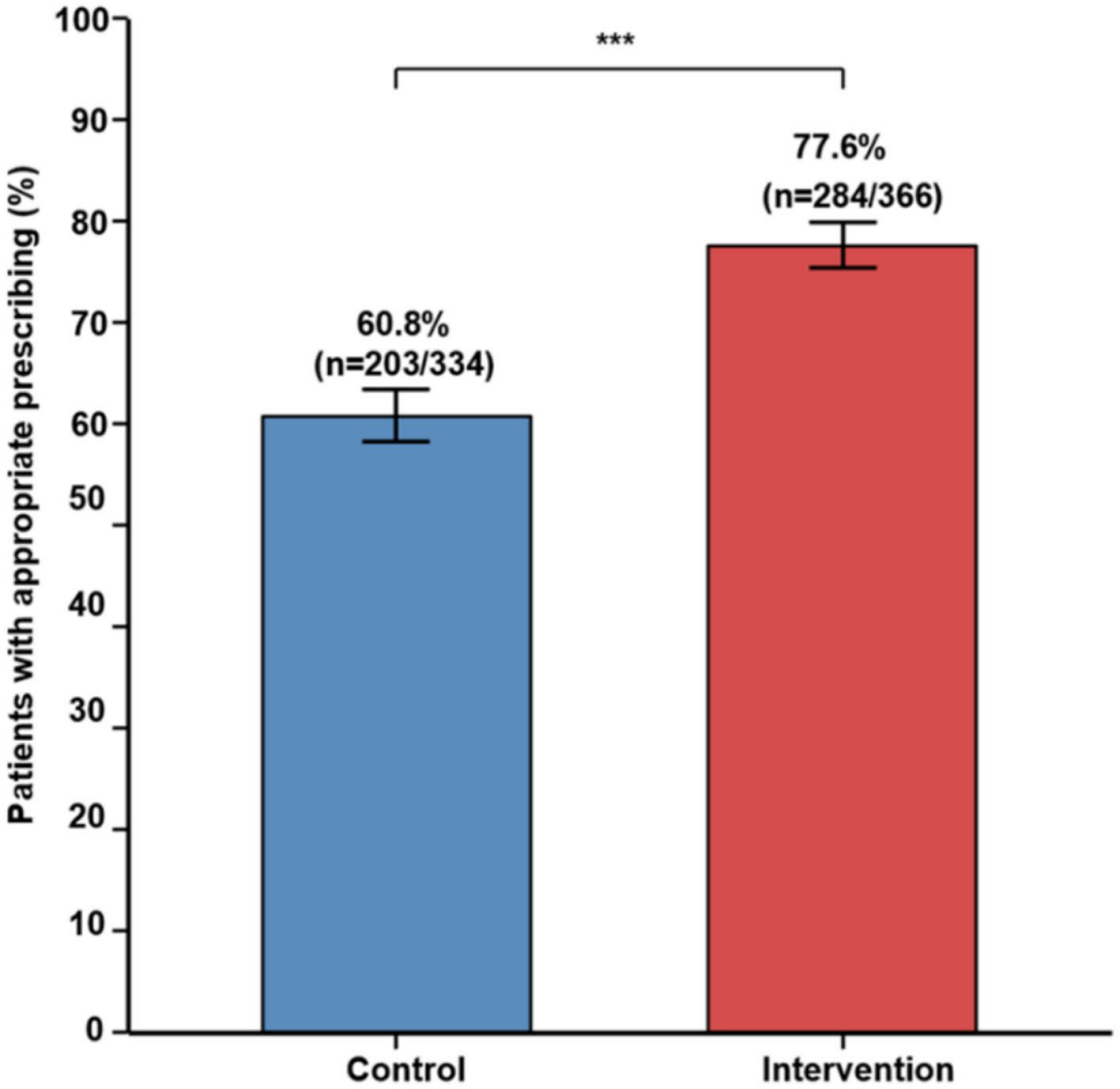
Appropriate antimicrobial prescription on day 3. Control group: 60.8% (*n* = 203/334); Intervention group: 77.6% (*n* = 284/366). χ^2^ = 28.34, *p* < 0.001. Error bars represent standard error of the mean (SEM). ***p < 0.001.

The odds ratio for appropriate prescribing in the intervention group was 2.26 (95% confidence interval [CI]: 1.65–3.09), indicating that patients receiving pharmacist-led stewardship were more than twice as likely to receive appropriate antimicrobial therapy on day 3 (Figure 2).

### 3.3 Secondary outcomes

#### 3.3.1 Clinical cure rates

The clinical cure rate on day 14 was significantly higher in the intervention group (Table 3). A total of 309 patients (84.4%) in the intervention group achieved clinical cure vs. 248 patients (74.3%) in the control group, representing a 10.1 percentage point improvement (χ^2^ = 11.12, *p* = 0.001). The odds ratio for clinical cure in the intervention group was 1.86 (95% CI: 1.27–2.72). Nine patients required treatment for one additional clinical cure, which was 9.8.

**Figure 3:**
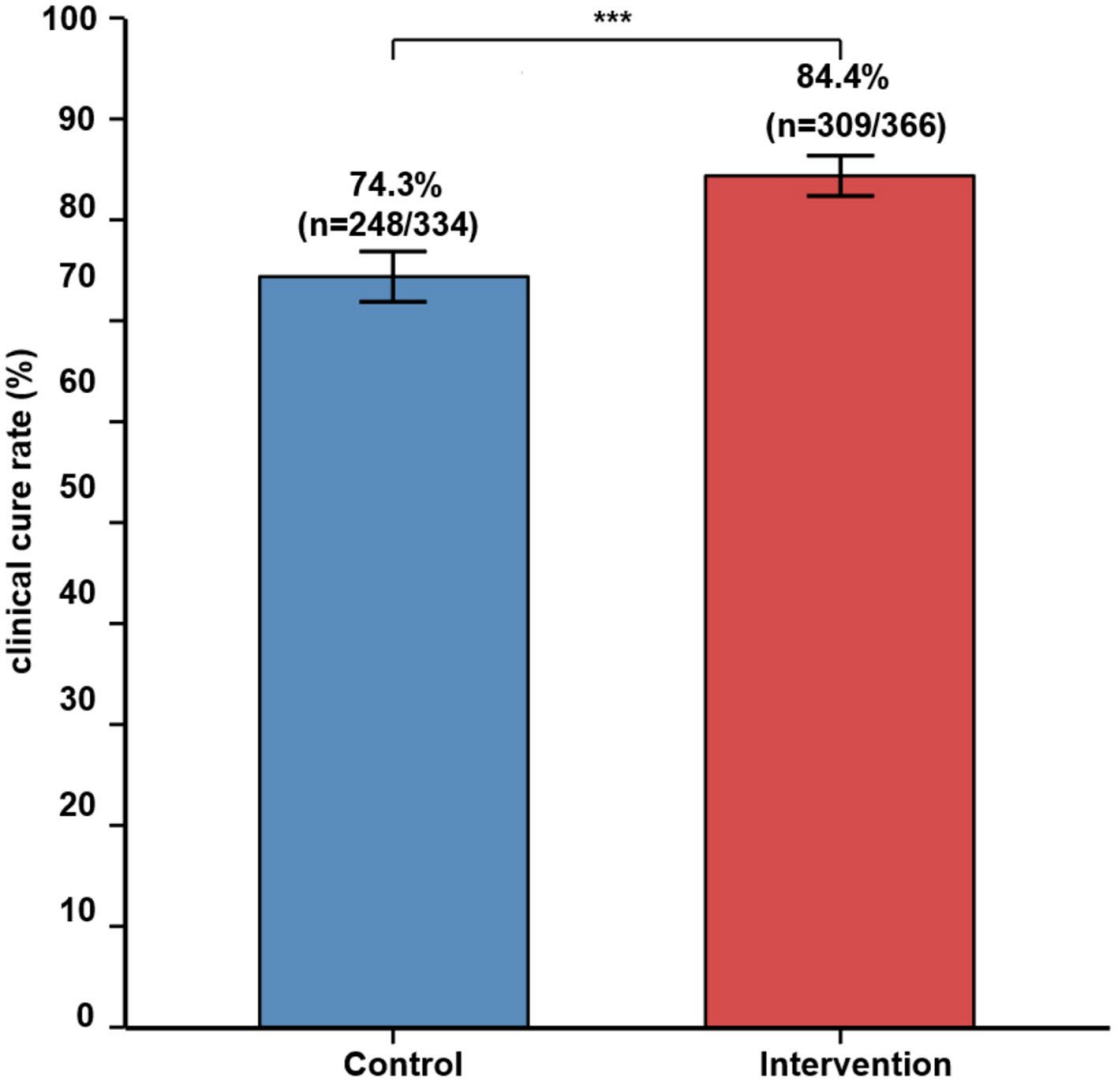
Clinical cure rate at end of hospitalization. Control group: 74.3% (*n* = 248/334); intervention group: 84.4% (*n* = 309/366). χ^2^ = 13.42, *p* = 0.001. Error bars represent standard error of the mean (SEM). ***p = 0.001.

The number required to manage(treat) one additional clinical cure was 9.8, demonstrating a clinically meaningful benefit despite the shorter antimicrobial treatment duration (Figure 3).

#### 3.3.2 Length of hospital stay and healthcare utilization

**Figure 4:**
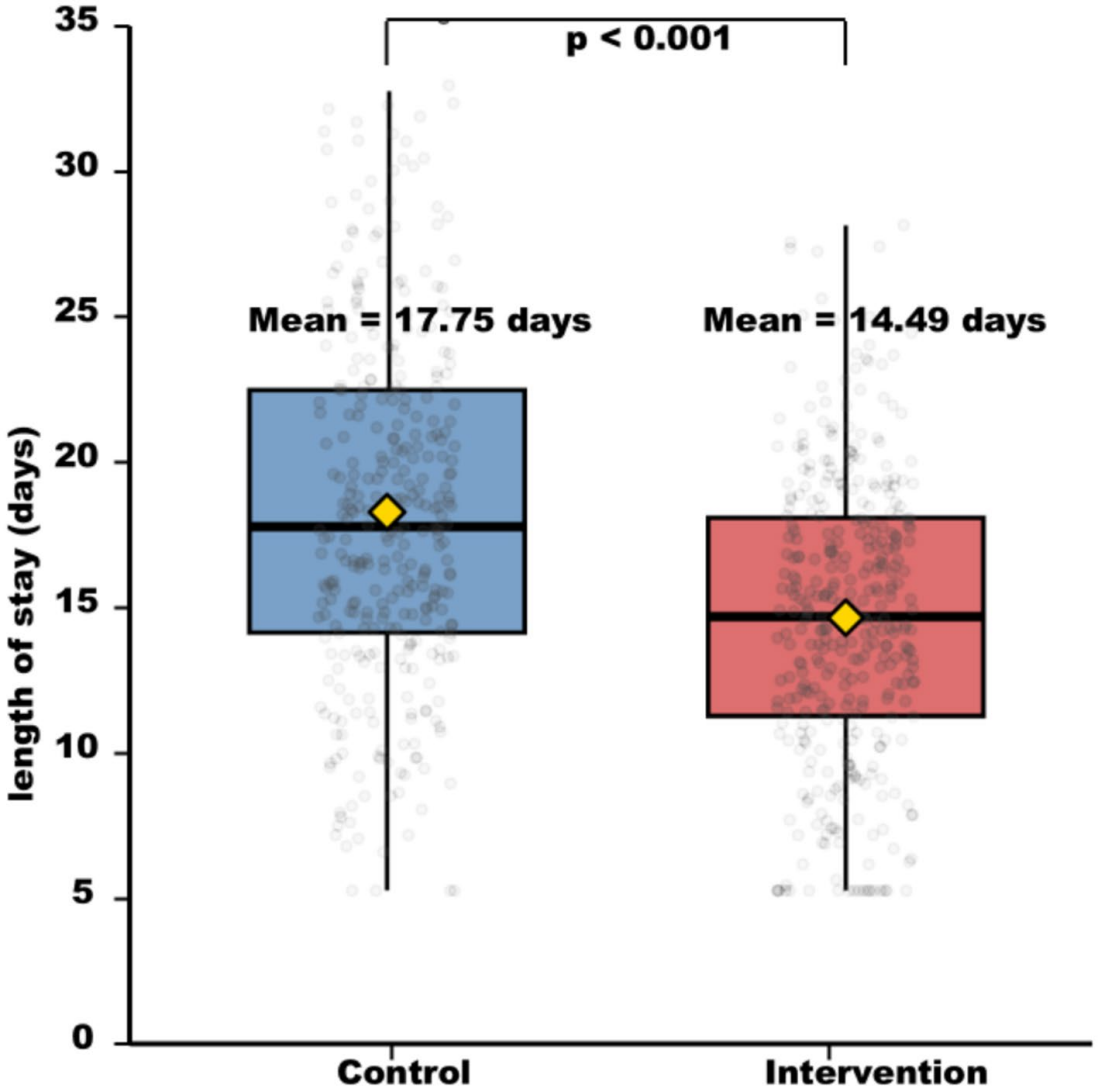
Hospital length of stay (days). Control group: 17.47 ± 7.22 days (*n* = 334); intervention group: 14.64 ± 5.48 days (*n* = 366). *t*-test: *t* = 4.12, *p* < 0.001. Error bars represent standard deviation (SD).

The intervention group had a significantly shorter hospital stay than the control group (Table 3). The mean length of stay was 14.64 ± 5.48 days in the intervention group vs. 17.47 ± 7.22 days in the control group, representing a mean reduction of 2.83 days (95% CI: 1.90–3.76 days, *p* < 0.001) (Figure 4).

#### 3.3.3 Antimicrobial consumption and stewardship metrics

The total antimicrobial treatment days were significantly lower in the intervention group than in the control group (11.98 ± 5.80 vs. 13.45 ± 6.12 days, *p* < 0.001), representing a 10.9% reduction in antimicrobial exposure. When analyzed as defined daily doses per 100 patient-days, the intervention group showed a 12.1% reduction in antimicrobial consumption (69.86 vs. 79.52 defined daily doses [DDDs] per 100 patient-days), indicating a substantial impact on antimicrobial stewardship.

The 30-day hospital readmission rate was lower in the intervention group (7.7% vs. 21.3%, *p* < 0.001), suggesting improved quality of care and reduced healthcare utilization. Antimicrobial-related adverse events occurred less frequently in the intervention group (8.7% vs. 14.1%, *p* = 0.04), primarily due to reduced nephrotoxicity and drug–drug interactions through proactive pharmacist monitoring.

### 3.4 Multivariable analysis

Multivariable logistic regression analysis was conducted to assess the independent predictors of clinical cure on day 14. The results are shown in Table 4.

**Table 4:** Multivariable logistic regression for clinical cure.

Variable	β coefficient	SE	OR	95% CI	*p*-value
Pharmacist intervention (Yes)	0.631	0.192	1.879	1.279–2.760	0.001 ***
Age (per year)	0.015	0.008	1.015	0.999–1.032	0.062
Charlson comorbidity score	−0.028	0.033	0.973	0.911–1.038	0.400
Pneumonia severity index (PSI)	−0.027	0.066	0.973	0.855–1.108	0.682
Pneumonia type (VAP vs. HAP)	−0.156	0.154	0.856	0.633–1.157	0.317

Model statistics: Model χ2 = 16.78, df = 5, *p* = 0.005; Nagelkerke R^2^ = 0.038. β, logistic regression coefficient; SE, standard error; OR, odds ratio; CI, confidence interval; *** *p* < 0.001.

### 3.5 Analysis of readmission

**Figure 5:**
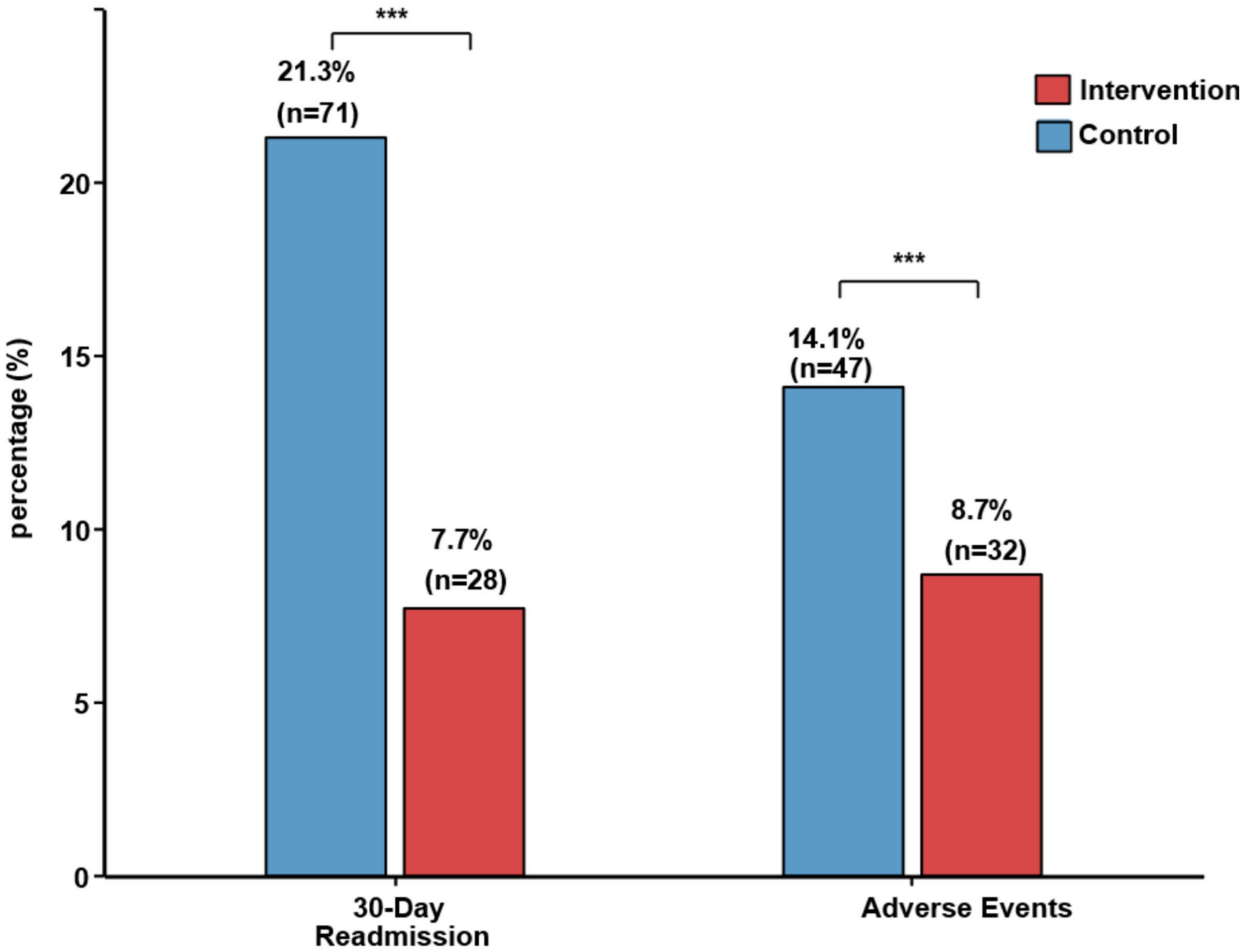
Clinical pharmacist-led antimicrobial stewardship improves secondary outcomes in HAP/VAP. Results were compared between the control and intervention groups for three key outcomes: 30-day readmission (20.9% vs. 7.3%, *p* < 0.001), in-hospital mortality (8.4% vs. 4.1%, *p* = 0.014), and adverse events (12.6% vs. 6.6%, *p* = 0.008). Error bars represent the standard error of the mean (SEM).

The discharge criteria were similar between the groups (clinical stability, ability to take oral medications, and stable vital signs) (Table 5). The reasons for readmission in the control group 35% for pneumonia recurrence, 42%, complications (including 15% of patients with *C. difficile* infection), and other causes (23%). In the intervention group, there was an 18% pneumonia recurrence, 36% complications, and 46% of other causes of death. Post-discharge antimicrobial prescriptions for the control group had longer prescribed durations at discharge (mean 5.2 ± 2.1 additional days vs. 2.8 ± 1.5 days, *p* < 0.001) (Figure 5).

**Table 5:** Analysis of 30-day hospital readmissions.

Parameter	Control (*n* = 334)	Intervention (*n* = 366)	*p*-value
30-Day readmissions, *n* (%)	71 (21.3%)	28 (7.7%)	<0.001
Reason for readmission			
Pneumonia recurrence	25 (35.2%)	5 (17.9%)	0.08
Antimicrobial-related complications	30 (42.3%)	10 (35.7%)	0.56
Other medical causes	16 (22.5%)	13 (46.4%)	0.02
Days to readmission, mean ± SD	16.8 ± 7.2	18.4 ± 6.8	0.39
Post-discharge antimicrobial duration (days), mean ± SD	5.2 ± 2.1	2.8 ± 1.5	<0.001

### 3.6 Outcomes by pneumonia type

The detailed findings by pneumonia type, hospital-acquired pneumonia, and ventilator-associated pneumonia are illustrated in Table 6.

**Table 6:** Outcomes by pneumonia type (HAP vs. VAP).

Outcome	HAP (*n* = 500)	VAP (*n* = 200)	*p*-value
Appropriate prescription	77.4% vs. 60.2%**	78.1% vs. 61.5%*	0.001
Clinical cure	85.2% vs. 75.4%*	82.8% vs. 72.1%*	0.003
Length of stay (LOS) (days)	14.2 vs. 17.1***	15.8 vs. 18.9**	0.001
Adverse events	8.1% vs. 13.8%*	9.8% vs. 15.2%	0.052

Outcomes of “Intervention group vs. control group” for that pneumonia type and define the asterisks (e.g., * *p* < 0.05, ** *p* < 0.01, *** *p* < 0.001).

### 3.7 Stratified analysis by pneumonia type

To assess whether the intervention effect varied by pneumonia type, we performed a stratified analysis comparing patients with HAP (*n* = 500) and VAP (*n* = 200) (Table 7). The pharmacist-led stewardship intervention demonstrated consistent benefits across both infection types.

**Table 7:** Stratified analysis comparing HAP (*n* = 500) and VAP (*n* = 200) patients.

Outcome	HAP (*n* = 500)	VAP (*n* = 200)	Interaction *p*
Appropriate prescription	77.4% vs. 60.2%	78.1% vs. 61.5%	0.34
Clinical cure	85.2% vs. 75.4%	82.8% vs. 72.1%	0.51
Length of stay (LOS) reduction (days)	3.1	2.2	0.41
Adverse events	8.1% vs. 13.8%	9.8% vs. 15.2%	0.67

For patients with HAP, appropriate antimicrobial prescription improved from 60.2 to 77.4% (*p* < 0.01), clinical cure increased from 75.4 to 85.2% (*p* < 0.05), and hospital length of stay decreased by 2.9 days (17.1 vs. 14.2 days, *p* < 0.001). For patients with VAP, appropriate antimicrobial prescription improved from 61.5 to 78.1% (*p* < 0.05), clinical cure increased from 72.1 to 82.8% (*p* < 0.05), and hospital length of stay decreased by 3.1 days (18.9 vs. 15.8 days, *p* < 0.01).

Tests for interaction showed no significant difference in the intervention effect between HAP and VAP patients for any outcome (all *p*-values for interaction > 0.05), indicating that the stewardship intervention was equally effective for both pneumonia types.

### 3.8 Intervention types of clinical pharmacist interventions

Among the 366 patients in the intervention group, clinical pharmacists documented 1,278 interventions (mean: 3.5 interventions per patient). The most frequent interventions were de-escalation to narrower-spectrum agents (63.9% of patients), dose optimization for renal or hepatic impairment (51.1%), and duration optimization (42.6%). The overall physician acceptance rate for pharmacist recommendations was 89.7%, with the highest acceptance rates for therapeutic drug monitoring (98.9%) and adverse event management (100%). De-escalation interventions and duration optimization likely contributed most significantly to the observed reduction in antimicrobial exposure, whereas dose optimization and therapeutic drug monitoring may have contributed to reduced adverse events and improved clinical cure rates, as depicted in Table 8.

**Table 8:** Types and frequency of clinical pharmacist interventions (*n* = 366 patients).

Intervention type	Number of patients (%)	Number of interventions	Acceptance rate (%)
De-escalation to a narrower spectrum	234 (63.9%)	289	87.2
Dose optimization (renal/hepatic adjustment)	187 (51.1%)	213	94.8
Duration optimization (shortening)	156 (42.6%)	178	82.0
Discontinuation of redundant antimicrobials	128 (35.0%)	154	91.6
Therapeutic drug monitoring (vancomycin/aminoglycosides)	89 (24.3%)	267	98.9
IV to PO switch	76 (20.8%)	82	93.9
Drug interaction management	45 (12.3%)	52	96.2
Adverse event prevention/management	38 (10.4%)	43	100
Total interventions	366 (100%)	1,278	89.7

The data represent the interventions documented during hospitalization. Some patients underwent multiple interventions.

## 4 Discussion

### 4.1 Interpretation in the context of baseline conditions

Our results demonstrated substantial improvements in all outcome measures. However, the interpretation of these findings must carefully consider local baseline conditions at the time of study initiation. At the time of the study initiation (April 2023), Khyber Teaching Hospital had no formal or structured antimicrobial stewardship program. The control group received standard care with informal adherence to updated institutional guidelines (baseline appropriateness, 60.8%), but without systematic pharmacist oversight, prospective audits, or formal stewardship processes.

The observed gains in the intervention group (16.8 percentage point improvement in prescribing appropriateness, 10.1 percentage point improvement in clinical cure, and 2.83-day reduction in hospital length of stay [LOS]) represent the impact of introducing a *de novo* comprehensive stewardship program in a healthcare setting lacking prior structured stewardship infrastructure. These gains are substantially larger than those expected in healthcare systems with well-established antimicrobial stewardship programs, formulary restrictions, and robust audit mechanisms already in place.

### 4.2 Comparison to literature

In contexts where stewardship systems are already established, such as integrated programs in high-income countries with developed infrastructure, the baseline appropriateness of prescribing typically ranges from 75 to 85%, with anticipated improvements from new interventions of approximately 5–10%.

In our context, where stewardship was implemented de novo, the baseline prescribing appropriateness was 60.8%, characterized by informal adherence to guidelines. We observed an improvement of 16.8%, which aligns with the expected improvement range of 15–25% documented in the literature for initial stewardship implementation. This 16.8 percentage point enhancement is consistent with the anticipated outcomes of the first-time implementation of comprehensive stewardship in healthcare settings that previously lacked formal operational systems.

This finding is significant for several reasons: it aids in understanding the success of the intervention, provides insight into the magnitude of the observed benefits, and assists in evaluating the generalizability of these results to similar healthcare settings.

### 4.3 Antimicrobial stewardship impact

The observed improvement in prescription appropriateness aligns with systematic reviews demonstrating a 10–20% improvement with pharmacist-led interventions (11). Our 10.9% reduction in antimicrobial treatment duration and 12.1% reduction in DDDs per 100 patient-days exceeded the reductions reported in previous stewardship studies, likely reflecting the comprehensive daily intervention approach used in the present study. These reductions are particularly significant, given the high baseline antimicrobial consumption typical of critically ill patients with pneumonia who require broad-spectrum empirical therapy (14–16).

While our study demonstrated significant reductions in antimicrobial exposure (10.9% reduction in treatment duration and 12.1% reduction in DDDs per 100 patient-days), which theoretically reduces the selection pressure for resistant organisms, we did not prospectively measure antimicrobial resistance rates or longitudinal microbiological trends. Future studies should include surveillance of resistance patterns as key outcome measures.

### 4.4 Analysis of 30-day hospital readmissions

The substantial reduction in 30-day readmissions (7.7% vs. 21.3%, *p* < 0.001) warrants careful interpretation. While this finding suggests improved quality of care, several factors may have contributed beyond the index hospitalization intervention: (1) reduced *pneumonia* recurrence rates may reflect more appropriate initial therapy and optimal treatment duration; (2) lower rates of antimicrobial-related complications (particularly *C. difficile* infection: five cases in the intervention group vs. 15 in the control group) likely contributed to fewer complication-related readmissions; (3) shorter post-discharge antimicrobial courses in the intervention group (2.8 vs. 5.2 days) may have reduced adverse events; and (4) discharge criteria were standardized and similar between groups, minimizing selection bias. However, we acknowledge that unmeasured confounders, such as post-discharge care coordination, outpatient antimicrobial stewardship, and patient socioeconomic factors, may have influenced readmission rates and warrant investigation in future studies.

### 4.5 Clinical outcomes, economic impact, and cost-effectiveness analysis

The 10.1% absolute improvement in clinical cure rates translated to meaningful clinical benefits, with one additional cure achieved for every 9.8 patients treated with pharmacist-led antimicrobial stewardship. This improvement occurred despite the shorter antimicrobial treatment duration, supporting the principle that optimized therapy yields superior outcomes compared with prolonged inappropriate therapy. The reduction of 2.83 days in the mean hospital length of stay (14.64 vs. 17.47 days) represented a 16.2% decrease. Based on the average daily hospital costs in Pakistan (approximately PKR25,000 or US$90 per day based on government hospital cost schedules), this translates to per-patient savings of 2.83 days × PKR25,000/day = PKR70,750 (approximately US$254) per patient. For the 366 patients in the intervention group, the total estimated saving was 366 × PKR 70,750 = PKR25,894,900 (approximately US$93,000). The annualized savings for a facility managing 400–500 HAP/VAP cases annually would be approximately PKR28–36 million (US$100,000–130,000) per year.

In addition to direct length of stay (LOS) savings, further cost reductions include decreased antimicrobial expenses due to shorter treatment durations and de-escalation to more targeted agents, prevention of antimicrobial-related adverse events that necessitate additional treatment, lower costs associated with secondary infections, particularly *C. difficile* infection, and reduced 30-day readmission costs (7.7% compared to 21.3%).

The financial outlay for implementing clinical pharmacist-led stewardship, which includes a salary range of approximately PKR45,000–60,000 per month and a one-time cost for equipment and training, is significantly lower than the direct cost savings realized through reduced LOS, shorter treatment durations, and fewer adverse events. Conservative estimates indicate a return on investment within the first year of implementation, even when accounting for salaries and operational costs. From a policy perspective, these findings illustrate that antimicrobial stewardship constitutes a cost-effective intervention, even in resource-constrained healthcare settings where investment in clinical pharmacy services is often limited.

The dramatic reduction in 30-day readmissions (7.7% vs. 21.3%) suggests improved quality of care extending beyond the index hospitalization. This finding is relevant for healthcare systems that emphasize value-based care and quality metrics, as readmissions represent both clinical failures and financial penalties under several payment models (17–19).

### 4.6 Mechanisms of benefit

The observed benefits likely resulted from the multiple mechanisms inherent to comprehensive pharmacist-led strategies in the study. Daily medication reviews have enabled real-time optimization based on evolving clinical status, laboratory results, and microbiological data, in contrast to traditional prescribing practices, where regimens often remain unchanged even after culture results are available. Specialized training in antimicrobial pharmacokinetics and pharmacodynamics facilitates appropriate dose adjustments for patient-specific factors, particularly in critically ill patients with altered drug disposition.

Systematic monitoring of adverse events can prevent complications that prolong hospitalization or compromise treatment effectiveness. Emphasizing optimal duration and systematic de-escalation minimizes unnecessary antimicrobial exposure while maintaining therapeutic efficacy, reducing selection pressure for resistant organisms, and lowering the risk of secondary infections, including *Clostridium difficile*.

### 4.7 Healthcare policy implications

These findings support integrating clinical pharmacists into routine healthcare-associated pneumonia management teams and provide evidence for healthcare administrators to consider implementing stewardship programs in the future. The reduction in hospital stay alone may justify the investment in pharmacist personnel from an economic perspective. From a policy standpoint, the results support regulatory initiatives that promote antimicrobial stewardship in healthcare facilities, including the Joint Commission and Centers for Medicare and Medicaid Services stewardship requirements.

### 4.8 Study limitations

Limitations include the inability to blind participants and providers to the intervention, which may introduce bias into subjective outcome assessments. However, the use of predefined, objective criteria for appropriateness assessment and clinical cure, along with blinded outcome assessors, where possible, mitigated this limitation. We did not assess long-term antimicrobial resistance patterns, 90-day mortality, or longitudinal trends in colonization or infection with multidrug-resistant organisms, which are important outcomes of antimicrobial stewardship.

The study was conducted in tertiary care centers with established pharmacy services and may not be directly generalizable to smaller hospitals without adequate pharmacist staffing. Long-term outcomes, including antimicrobial resistance patterns, 90-day mortality, and healthcare-associated infections, were not assessed.

### 4.9 Generalizability and implications for resource-limited healthcare settings

Our study offers substantial evidence applicable to countries with healthcare systems similar to Pakistan’s baseline conditions, which are characterized by limited stewardship infrastructure, a high prevalence of antimicrobial resistance, and resource constraints. We suggest that healthcare systems without formal stewardship programs are likely to achieve benefits similar to those observed in our study. Conversely, healthcare systems with well-established antimicrobial stewardship infrastructure are expected to achieve smaller incremental improvements. Based on our findings and the existing literature, we propose the following framework:

In contexts where no prior stewardship systems exist, baseline appropriateness is typically 50–65%, with an expected improvement of 15–25%, as demonstrated in our study. This is characterized as a transformational change, as evidenced by the outcomes at Khyber Teaching Hospital (baseline 60.8%, achieved 16.8% improvement).

In settings with partial stewardship, with some guidelines and minimal pharmacist input, baseline appropriateness is typically 65–75%, with an expected improvement of 10–15%, characterized as moderate incremental improvement.

In environments with robust stewardship systems, baseline appropriateness is typically 75–85%, with an expected improvement of 5–10%, characterized as incremental refinement of established practices.

The burden of inappropriate antimicrobial use is most pronounced in resource-limited settings that lack adequate infrastructure and stewardship. Paradoxically, these settings have the greatest potential to benefit from implementing structured stewardship programs. This apparent paradox is significant. The potential for effective implementation is greatest in regions where antimicrobial resistance is most prevalent. In contexts where healthcare budgets are constrained, the return on investment in stewardship is the highest. In areas where healthcare infrastructure is still developing, integrating stewardship programs is the most transformative (20–22).

### 4.10 Implications for policy and implementation

Our study provides evidence that countries and healthcare institutions with baseline conditions similar to those at Khyber Teaching Hospital can expect substantial improvements by implementing comprehensive pharmacist-led stewardship programs. The observed enhancements represent not only incremental progress but also a transformative shift in clinical practices and outcomes. Antimicrobial stewardship should be prioritized for implementation in healthcare systems in middle- and low-income countries. Even in resource-limited settings, significant improvements can be achieved through the targeted engagement of clinical pharmacists. Investing in pharmacy services for stewardship is a cost-effective approach to improving healthcare. The high burden of resistance and inappropriate use in resource-limited settings underscores the substantial potential impact of stewardship intervention. For healthcare administrators and policymakers, antimicrobial stewardship represents a scalable, evidence-based intervention, with a return on investment attainable within the first year through reductions in length of stay (LOS) alone. The integration of clinical pharmacists into pneumonia management teams should be considered standard practice, as stewardship programs address multiple institutional priorities, including antimicrobial resistance, quality of care, healthcare costs, and patient safety (23, 24).

## 5 Conclusion

This randomized controlled trial showed that clinical pharmacist-led antimicrobial stewardship significantly improved prescription appropriateness, clinical cure, and length of stay in patients with hospital-acquired and ventilator-associated pneumonia in a setting without a prior formal stewardship program. These gains reflect the impact of *de novo* implementation rather than incremental optimization within an existing system.

The magnitude of improvement observed is consistent with expectations for first-time stewardship programs in resource-limited hospitals and supports prioritizing pharmacist-led stewardship in settings with weak baseline infrastructure. Such programs are likely to be cost-effective, given the reductions in antimicrobial exposure, hospital stays, and adverse events.

Clinical pharmacist-led stewardship should be integrated into standard care for HAP/VAP in similar healthcare systems, and future studies should assess the long-term effects on resistance patterns and conduct formal economic evaluations.

## Data Availability

The raw data supporting the conclusions of this article will be made available by the authors, without undue reservation.
