# Supplementary material for: Impact of clinical pharmacist-led antimicrobial stewardship on antibiotic appropriateness, clinical outcomes, and antimicrobial consumption in hospital-acquired and ventilator-associated pneumonia: a randomized controlled trial
Source: Front Public Health. 2026 Apr 7;14:1700328. doi: 10.3389/fpubh.2026.1700328 (PMC13095787; doi:10.3389/fpubh.2026.1700328)
Supplement: Supplementary file 1 [file Data_Sheet_1.PDF]

```
DATASET ACTIVATE DataSet1.
```

Dataset Activate

Notes

Output Created		30-AUG-2025 17:31:55
Comments		
Input	Filter	<none>
	Weight	<none>
	Split File	<none>
Syntax		DATASET ACTIVATE DataSet1.
Resources	Processor Time	00:00:00.00
	Elapsed Time	00:00:00.00

```
GET DATA
  /TYPE=TXT
  /FILE="C:/Users/zshah/Desktop/depart annual eEaluation 2024-25\Dr.ilyas project\hospital me
  /DELCASE=LINE
  /DELIMITERS=","
  /QUALIFIER='"'
  /ARRANGEMENT=DELIMITED
  /FIRSTCASE=2
  /VARIABLES=
    patient_id F8
    group F1
    age F8
    charlson_score F8
    psi_score F8
    appropriate F1
    clinical_cure F1
    LOS F8
    antibiotic_days F8
    readmission30 F1
    adverse_event F1.
CACHE.
EXECUTE.

VARIABLE LABELS
 group 1 'Control' 2 'Intervention'
  /appropriate 0 'No' 1 'Yes'
  /clinical_cure 0 'No' 1 'Yes'
  /readmission30 0 'No' 1 'Yes'
  /adverse_event 0 'No' 1 'Yes'.

VARIABLE LABELS
  patient_id 'Patient ID'
  group 'Study Group'
```

```
  age 'Age (years)'
  charlson_score 'Charlson Comorbidity Index'
  psi_score 'Pneumonia Severity Index'
  appropriate 'Appropriate Antibiotic Prescribing (Day 3)'
  clinical_cure 'Clinical Cure (Day 14)'
  LOS 'Length of Hospital Stay (days)'
  antibiotic_days 'Total Antibiotic Treatment Days'
  readmission30 '30-Day Hospital Readmission'
  adverse_event 'Antibiotic-Related Adverse Events'.

DESCRIPTIVES VARIABLES=age charlson_score psi_score LOS antibiotic_days
  /STATISTICS=MEAN STDDEV MIN MAX.
```

Descriptives

Notes

Output Created		30-AUG-2025 17:31:56
Comments		
Input	Data	C:\Users\zshah\Desktop\depart annual ealuation 2024-25\Dr.ilyas project\hospital medication\asp_study_700.csv
	Filter	<none>
	Weight	<none>
	Split File	<none>
	N of Rows in Working Data File	700
Missing Value Handling	Definition of Missing	User defined missing values are treated as missing.
	Cases Used	All non-missing data are used.
Syntax		DESCRIPTIVES VARIABLES=age charlson_score psi_score LOS antibiotic_days /STATISTICS=MEAN STDDEV MIN MAX.
Resources	Processor Time	00:00:00.02
	Elapsed Time	00:00:00.02

Descriptive Statistics

	N	Minimum	Maximum	Mean	Std. Deviation
Age (years)	700	45	85	65.17	11.960
Charlson Comorbidity Index	700	0	9	4.48	2.913
Pneumonia Severity Index	700	1	5	3.07	1.434
Length of Hospital Stay (days)	700	4	29	15.99	6.518
Total Antibiotic Treatment Days	700	2	24	11.98	5.796
Valid N (listwise)	700				

```
 SPLIT FILE SEPARATE BY group.
DESCRIPTIVES VARIABLES=age charlson_score psi_score LOS antibiotic_days
  /STATISTICS=MEAN STDDEV MIN MAX.
```

Descriptives

Notes

Output Created		30-AUG-2025 17:31:56
Comments		
Input	Data	C: \Users\zshah\Desktop\depart annual ealuation 2024-25\Dr.ilyas project\hospital medication\asp_study_700.csv
	Filter	<none>
	Weight	<none>
	Split File	Study Group
	N of Rows in Working Data File	700
Missing Value Handling	Definition of Missing	User defined missing values are treated as missing.
	Cases Used	All non-missing data are used.
Syntax		DESCRIPTIVES VARIABLES=age charlson_score psi_score LOS antibiotic_days /STATISTICS=MEAN STDDEV MIN MAX.
Resources	Processor Time	00:00:00.36
	Elapsed Time	00:00:00.41

Warnings

The file is not sorted in a consistent manner on the split file variables. It is likely that any procedure output will be incorrect.

Study Group = 1

Descriptive Statistics[a]

	N	Minimum	Maximum	Mean	Std. Deviation
Age (years)	1	76	76	76.00	.
Charlson Comorbidity Index	1	7	7	7.00	.
Pneumonia Severity Index	1	5	5	5.00	.
Length of Hospital Stay (days)	1	5	5	5.00	.
Total Antibiotic Treatment Days	1	17	17	17.00	.
Valid N (listwise)	1				

a. Study Group = 1

Study Group = 2

Descriptive Statistics[a]

	N	Minimum	Maximum	Mean	Std. Deviation
Age (years)	2	58	68	63.00	7.071
Charlson Comorbidity Index	2	0	9	4.50	6.364
Pneumonia Severity Index	2	1	4	2.50	2.121
Length of Hospital Stay (days)	2	12	18	15.00	4.243
Total Antibiotic Treatment Days	2	6	10	8.00	2.828
Valid N (listwise)	2				

a. Study Group = 2

```
SPLIT FILE OFF.

FREQUENCIES VARIABLES=group appropriate clinical_cure readmission30 adverse_event
  /ORDER=ANALYSIS.
```

Frequencies

Notes

Output Created		30-AUG-2025 17:31:57
Comments		
Input	Data	C:<br>\Users\zshah\Desktop\depart annual ealuation 2024-25\Dr.ilyas project\hospital medication\asp_study_700.csv
	Filter	<none>
	Weight	<none>
	Split File	<none>
	N of Rows in Working Data File	700
Missing Value Handling	Definition of Missing	User-defined missing values are treated as missing.
	Cases Used	Statistics are based on all cases with valid data.
Syntax		FREQUENCIES VARIABLES=group appropriate clinical_cure readmission30 adverse_event<br>/ORDER=ANALYSIS.
Resources	Processor Time	00:00:00.02
	Elapsed Time	00:00:00.02

Statistics

		Study Group	Appropriate Antibiotic Prescribing (Day 3)	Clinical Cure (Day 14)	30-Day Hospital Readmission	Antibiotic-Related Adverse Events
N	Valid	700	700	700	700	700
	Missing	0	0	0	0	0

Frequency Table

Study Group

		Frequency	Percent	Valid Percent	Cumulative Percent
Valid	1	334	47.7	47.7	47.7
	2	366	52.3	52.3	100.0
	Total	700	100.0	100.0	

Appropriate Antibiotic Prescribing (Day 3)

		Frequency	Percent	Valid Percent	Cumulative Percent
Valid	0	213	30.4	30.4	30.4
	1	487	69.6	69.6	100.0
	Total	700	100.0	100.0	

Clinical Cure (Day 14)

		Frequency	Percent	Valid Percent	Cumulative Percent
Valid	0	143	20.4	20.4	20.4
	1	557	79.6	79.6	100.0
	Total	700	100.0	100.0	

30-Day Hospital Readmission

		Frequency	Percent	Valid Percent	Cumulative Percent
Valid	0	601	85.9	85.9	85.9
	1	99	14.1	14.1	100.0
	Total	700	100.0	100.0	

Antibiotic-Related Adverse Events

		Frequency	Percent	Valid Percent	Cumulative Percent
Valid	0	621	88.7	88.7	88.7
	1	79	11.3	11.3	100.0
	Total	700	100.0	100.0	

```
CROSSTABS
  /TABLES=group BY appropriate
  /FORMAT=AVALUE TABLES
  /STATISTICS=CHISQ
  /CELLS=COUNT ROW COLUMN TOTAL.
```

Crosstabs

Notes

Output Created		30-AUG-2025 17:31:57
Comments		
Input	Data	C:\Users\zshah\Desktop\depart annual ealuation 2024-25\Dr.ilyas project\hospital medication\asp_study_700.csv
	Filter	<none>
	Weight	<none>
	Split File	<none>
	N of Rows in Working Data File	700
Missing Value Handling	Definition of Missing	User-defined missing values are treated as missing.
	Cases Used	Statistics for each table are based on all the cases with valid data in the specified range(s) for all variables in each table.
Syntax		CROSSTABS /TABLES=group BY appropriate /FORMAT=AVALUE TABLES /STATISTICS=CHISQ /CELLS=COUNT ROW COLUMN TOTAL.
Resources	Processor Time	00:00:00.02
	Elapsed Time	00:00:00.01
	Dimensions Requested	2
	Cells Available	174762

Case Processing Summary

	Cases					
	Valid		Missing		Total	
	N	Percent	N	Percent	N	Percent
Study Group * Appropriate Antibiotic Prescribing (Day 3)	700	100.0%	0	0.0%	700	100.0%

Study Group * Appropriate Antibiotic Prescribing (Day 3) Crosstabulation

			Appropriate Antibiotic Prescribing (Day 3)		
			0	1	Total
Study Group	1	Count	131	203	334
		% within Study Group	39.2%	60.8%	100.0%
		% within Appropriate Antibiotic Prescribing (Day 3)	61.5%	41.7%	47.7%
		% of Total	18.7%	29.0%	47.7%
	2	Count	82	284	366
		% within Study Group	22.4%	77.6%	100.0%
		% within Appropriate Antibiotic Prescribing (Day 3)	38.5%	58.3%	52.3%
		% of Total	11.7%	40.6%	52.3%
Total		Count	213	487	700
		% within Study Group	30.4%	69.6%	100.0%
		% within Appropriate Antibiotic Prescribing (Day 3)	100.0%	100.0%	100.0%
		% of Total	30.4%	69.6%	100.0%

Chi-Square Tests

	Value	df	Asymp. Sig. (2-sided)	Exact Sig. (2-sided)	Exact Sig. (1-sided)
Pearson Chi-Square	23.330[a]	1	.000		
Continuity Correction[b]	22.543	1	.000		
Likelihood Ratio	23.446	1	.000		
Fisher's Exact Test				.000	.000
Linear-by-Linear Association	23.297	1	.000		
N of Valid Cases	700				

a. 0 cells (0.0%) have expected count less than 5. The minimum expected count is 101.63.

b. Computed only for a 2x2 table

```
LOGISTIC REGRESSION
 /DEPENDENT appropriate
 /METHOD=ENTER group
 /PRINT=CI(95)
 /CRITERIA=PIN(.05) POUT(.10) ITERATE(20) CUT(.5).
```

Logistic Regression

Notes

Output Created		30-AUG-2025 17:31:57
Comments		
Input	Data	C:<br>\Users\zshah\Desktop\depart annual ealuation 2024-25\Dr.ilyas project\hospital medication\asp_study_700.csv
	Filter	<none>
	Weight	<none>
	Split File	<none>
	N of Rows in Working Data File	700
Syntax		LOGISTIC REGRESSION<br>/DEPENDENT appropriate<br>/METHOD=ENTER group<br>/PRINT=CI(95)<br>/CRITERIA=PIN(.05) POUT(.10) ITERATE(20) CUT(.5).
Resources	Processor Time	00:00:00.00
	Elapsed Time	00:00:00.00

```
CROSSTABS
  /TABLES=group BY clinical_cure
  /FORMAT=AVALUE TABLES
  /STATISTICS=CHISQ
  /CELLS=COUNT ROW COLUMN TOTAL.
```

Crosstabs

Notes

Output Created		30-AUG-2025 17:31:57
Comments		
Input	Data	C:<br>\Users\zshah\Desktop\depart annual ealuation 2024-25\Dr.ilyas project\hospital medication\asp_study_700.csv
	Filter	<none>
	Weight	<none>
	Split File	<none>
	N of Rows in Working Data File	700
Missing Value Handling	Definition of Missing	User-defined missing values are treated as missing.
	Cases Used	Statistics for each table are based on all the cases with valid data in the specified range(s) for all variables in each table.
Syntax		CROSSTABS<br>/TABLES=group BY clinical_cure<br>/FORMAT=AVALUE TABLES<br>/STATISTICS=CHISQ<br>/CELLS=COUNT ROW COLUMN TOTAL.
Resources	Processor Time	00:00:00.00
	Elapsed Time	00:00:00.00
	Dimensions Requested	2
	Cells Available	174762

Case Processing Summary

	Cases					
	Valid		Missing		Total	
	N	Percent	N	Percent	N	Percent
Study Group * Clinical Cure (Day 14)	700	100.0%	0	0.0%	700	100.0%

Study Group * Clinical Cure (Day 14) Crosstabulation

			Clinical Cure (Day 14)		
			0	1	Total
Study Group	1	Count	86	248	334
		% within Study Group	25.7%	74.3%	100.0%
		% within Clinical Cure (Day 14)	60.1%	44.5%	47.7%
		% of Total	12.3%	35.4%	47.7%
	2	Count	57	309	366
		% within Study Group	15.6%	84.4%	100.0%
		% within Clinical Cure (Day 14)	39.9%	55.5%	52.3%
		% of Total	8.1%	44.1%	52.3%
Total		Count	143	557	700
		% within Study Group	20.4%	79.6%	100.0%
		% within Clinical Cure (Day 14)	100.0%	100.0%	100.0%
		% of Total	20.4%	79.6%	100.0%

Chi-Square Tests

	Value	df	Asymp. Sig. (2-sided)	Exact Sig. (2-sided)	Exact Sig. (1-sided)
Pearson Chi-Square	11.122[a]	1	.001		
Continuity Correction[b]	10.505	1	.001		
Likelihood Ratio	11.153	1	.001		
Fisher's Exact Test				.001	.001
Linear-by-Linear Association	11.106	1	.001		
N of Valid Cases	700				

a. 0 cells (0.0%) have expected count less than 5. The minimum expected count is 68.23.

b. Computed only for a 2x2 table

```
LOGISTIC REGRESSION
 /DEPENDENT clinical_cure
 /METHOD=ENTER group
 /PRINT=CI(95)
 /CRITERIA=PIN(.05) POUT(.10) ITERATE(20) CUT(.5).
```

Logistic Regression

Notes

Output Created		30-AUG-2025 17:31:57
Comments		
Input	Data	C:\Users\zshah\Desktop\depart annual ealuation 2024-25\Dr.ilyas project\hospital medication\asp_study_700.csv
	Filter	<none>
	Weight	<none>
	Split File	<none>
	N of Rows in Working Data File	700
Syntax		LOGISTIC REGRESSION /DEPENDENT clinical_cure /METHOD=ENTER group /PRINT=CI(95) /CRITERIA=PIN(.05) POUT(.10) ITERATE(20) CUT(.5).
Resources	Processor Time	00:00:00.00
	Elapsed Time	00:00:00.00

```
EXAMINE VARIABLES=LOS BY group
  /PLOT BOXPLOT
  /STATISTICS DESCRIPTIVES
  /CINTERVAL 95.
```

Explore

Notes

Output Created		30-AUG-2025 17:31:57
Comments		
Input	Data	C: \Users\zshah\Desktop\depart annual ealuation 2024-25\Dr.ilyas project\hospital medication\asp_study_700.csv
	Filter	<none>
	Weight	<none>
	Split File	<none>
	N of Rows in Working Data File	700
Missing Value Handling	Definition of Missing	User-defined missing values for dependent variables are treated as missing.
	Cases Used	Statistics are based on cases with no missing values for any dependent variable or factor used.
Syntax		EXAMINE VARIABLES=LOS BY group<br>/PLOT BOXPLOT<br>/STATISTICS DESCRIPTIVES<br>/CINTERVAL 95.
Resources	Processor Time	00:00:01.98
	Elapsed Time	00:00:01.45

Total Sample

Case Processing Summary

	Cases					
	Valid		Missing		Total	
	N	Percent	N	Percent	N	Percent
Length of Hospital Stay (days)	700	100.0%	0	0.0%	700	100.0%

Descriptives

			Statistic	Std. Error
Length of Hospital Stay (days)	Mean		15.99	.246
	95% Confidence Interval for Mean	Lower Bound	15.51	
		Upper Bound	16.47	
	5% Trimmed Mean		15.92	
	Median		16.00	
	Variance		42.491	
	Std. Deviation		6.518	
	Minimum		4	
	Maximum		29	
	Range		25	
	Interquartile Range		11	
	Skewness		.046	.092
	Kurtosis		-.945	.185

Length of Hospital Stay (days)

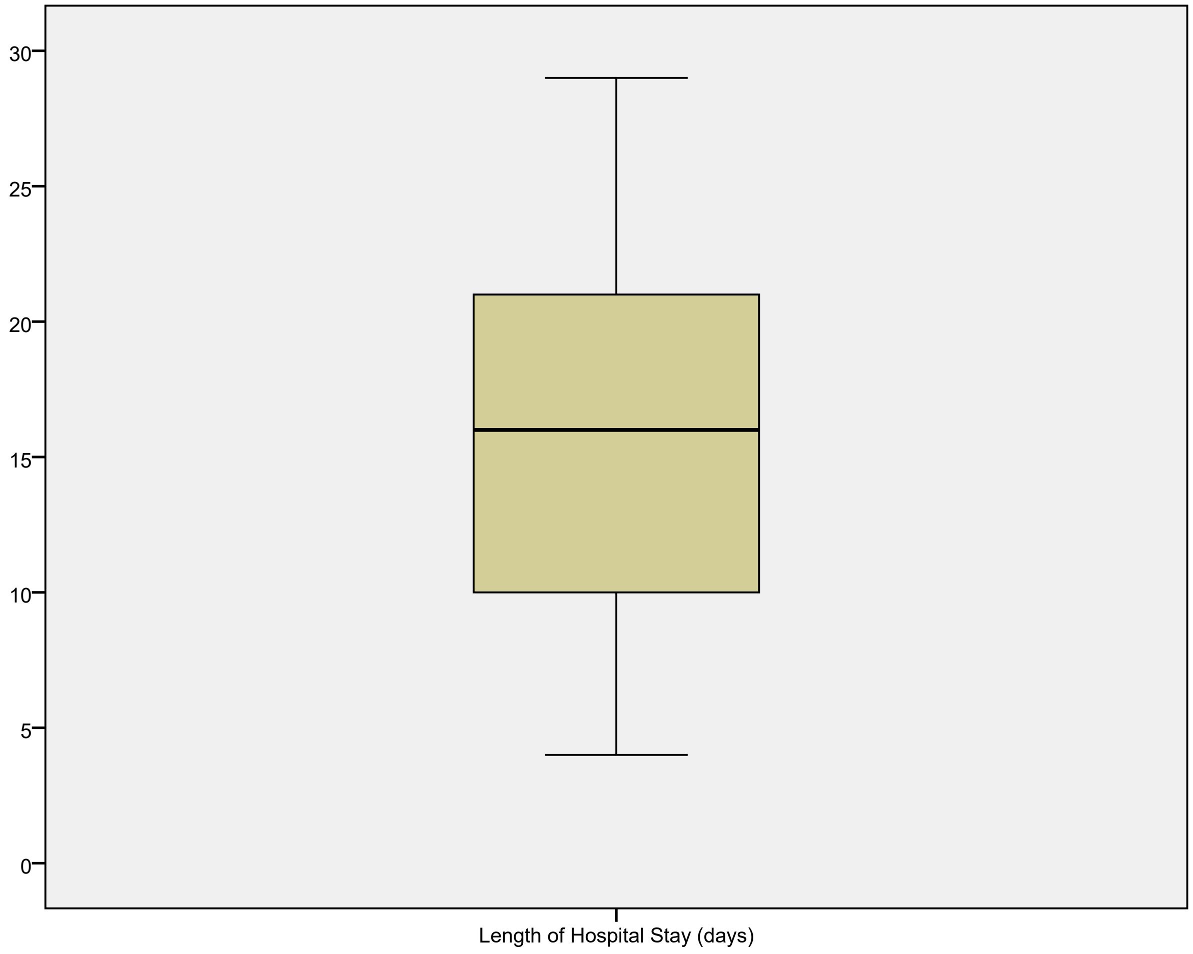

Study Group

Case Processing Summary

	Study Group	Cases				
		Valid		Missing		Total
		N	Percent	N	Percent	N
Length of Hospital Stay (days)	1	334	100.0%	0	0.0%	334
	2	366	100.0%	0	0.0%	366

Case Processing Summary

	Study Group	Cases
		Total
		Percent
Length of Hospital Stay (days)	1	100.0%
	2	100.0%

Descriptives

	Study Group			Statistic	Std. Error
Length of Hospital Stay (days)	1	Mean		17.47	.395
		95% Confidence Interval for Mean	Lower Bound	16.69	
			Upper Bound	18.24	
		5% Trimmed Mean		17.51	
		Median		18.00	
		Variance		52.100	
		Std. Deviation		7.218	
		Minimum		5	
		Maximum		29	
		Range		24	
		Interquartile Range		13	
		Skewness		-.100	.133
		Kurtosis		-1.190	.266
	2	Mean		14.64	.286
		95% Confidence Interval for Mean	Lower Bound	14.08	
			Upper Bound	15.21	
		5% Trimmed Mean		14.74	
		Median		15.00	
		Variance		30.022	
		Std. Deviation		5.479	
		Minimum		4	
		Maximum		23	
		Range		19	
		Interquartile Range		9	
		Skewness		-.192	.128
		Kurtosis		-1.115	.254

Length of Hospital Stay (days)

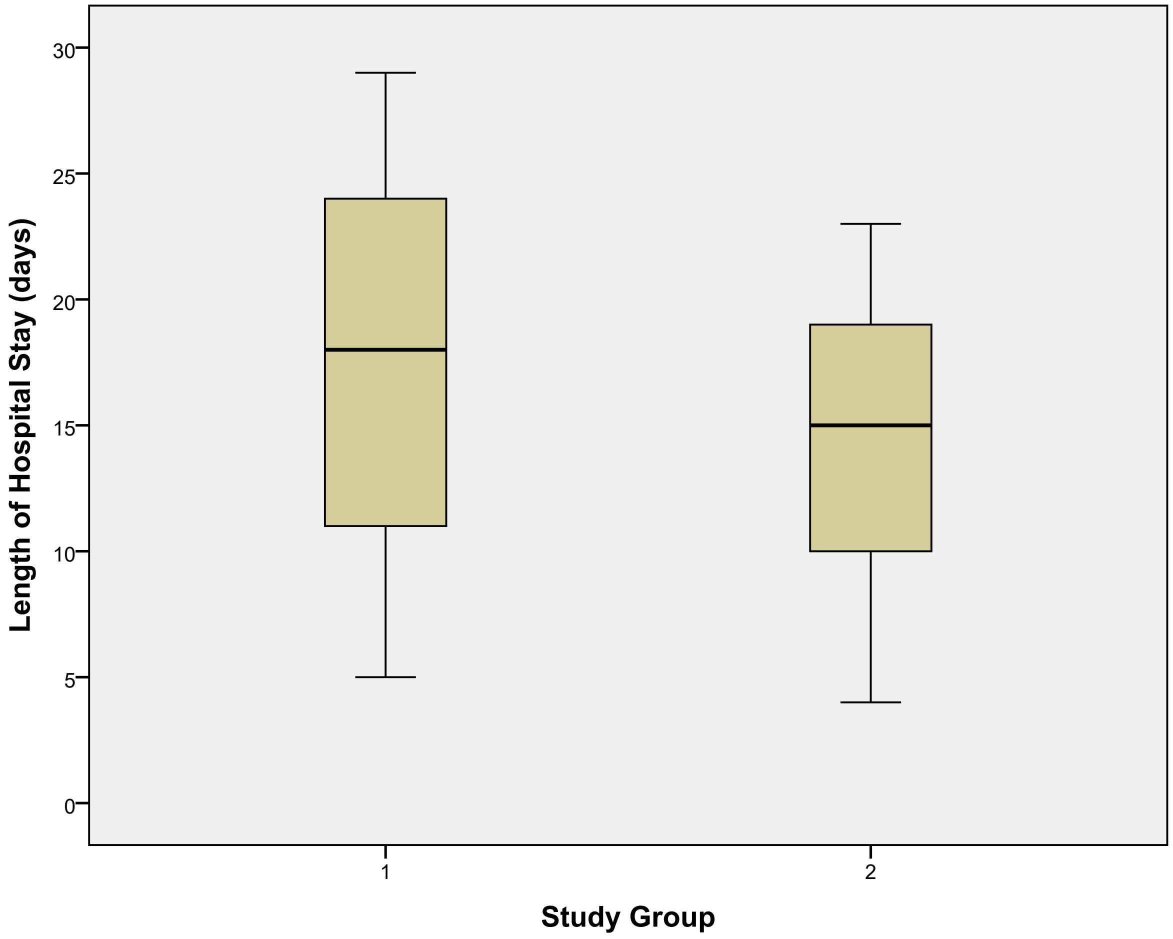


```
 T-TEST GROUPS=group(1 2)
/VARIABLES=LOS
 STATISTICS DESCRIPTIVES
 /CRITERIA=CI(.95).
```

T-Test

Notes

Output Created		30-AUG-2025 17:31:59
Comments		
Input	Data	C: \Users\zshah\Desktop\depart annual ealuation 2024-25\Dr.ilyas project\hospital medication\asp_study_700 .csv
	Filter	<none>
	Weight	<none>
	Split File	<none>
	N of Rows in Working Data File	700
Missing Value Handling	Definition of Missing	User defined missing values are treated as missing.
	Cases Used	Statistics for each analysis are based on the cases with no missing or out-of-range data for any variable in the analysis.
Syntax		T-TEST GROUPS=group (1 2) /VARIABLES=LOS STATISTICS DESCRIPTIVES /CRITERIA=CI(.95).
Resources	Processor Time	00:00:00.00
	Elapsed Time	00:00:00.00

```
T-TEST GROUPS=group(1 2)
 /VARIABLES=antibiotic_days
 /STATISTICS DESCRIPTIVES
 /CRITERIA=CI(.95).
```

T-Test

Notes

Output Created		30-AUG-2025 17:31:59
Comments		
Input	Data	C:<br>\Users\zshah\Desktop\depart annual ealuation 2024-25\Dr.ilyas project\hospital medication\asp_study_700.csv
	Filter	<none>
	Weight	<none>
	Split File	<none>
	N of Rows in Working Data File	700
Missing Value Handling	Definition of Missing	User defined missing values are treated as missing.
	Cases Used	Statistics for each analysis are based on the cases with no missing or out-of-range data for any variable in the analysis.
Syntax		T-TEST GROUPS=group(1 2)<br><br>/VARIABLES=antibiotic_days<br>/STATISTICS DESCRIPTIVES<br>/CRITERIA=CI(.95).
Resources	Processor Time	00:00:00.00
	Elapsed Time	00:00:00.00

Group Statistics

	Study Group	N	Mean	Std. Deviation	Std. Error Mean
Total Antibiotic Treatment Days	1	334	13.89	6.200	.339
	2	366	10.23	4.780	.250

Independent Samples Test

		Levene's Test for Equality of Variances		t-test for Equality of
		F	Sig.	t
Total Antibiotic Treatment Days	Equal variances assumed	26.538	.000	8.788
	Equal variances not assumed			8.686

Independent Samples Test

		t-test for Equality of Means		
		df	Sig. (2-tailed)	Mean Difference
Total Antibiotic Treatment Days	Equal variances assumed	698	.000	3.660
	Equal variances not assumed	624.551	.000	3.660

Independent Samples Test

		t-test for Equality of Means	
		Std. Error Difference	95% Confidence ...
			Lower
Total Antibiotic Treatment Days	Equal variances assumed	.416	2.842
	Equal variances not assumed	.421	2.832

Independent Samples Test

		t-test for Equality of ...
		95% Confidence ...
		Upper
Total Antibiotic Treatment Days	Equal variances assumed	4.477
	Equal variances not assumed	4.487

```
CROSSTABS
 /TABLES=group BY readmission30
 /FORMAT=AVALUE TABLES
 /STATISTICS=CHISQ
 /CELLS=COUNT ROW COLUMN TOTAL.
```

Crosstabs

Notes

Output Created		30-AUG-2025 17:31:59
Comments		
Input	Data	C:\Users\zshah\Desktop\depart annual ealuation 2024-25\Dr.ilyas project\hospital medication\asp_study_700.csv
	Filter	<none>
	Weight	<none>
	Split File	<none>
	N of Rows in Working Data File	700
Missing Value Handling	Definition of Missing	User-defined missing values are treated as missing.
	Cases Used	Statistics for each table are based on all the cases with valid data in the specified range(s) for all variables in each table.
Syntax		CROSSTABS /TABLES=group BY readmission30 /FORMAT=AVALUE TABLES /STATISTICS=CHISQ /CELLS=COUNT ROW COLUMN TOTAL.
Resources	Processor Time	00:00:00.02
	Elapsed Time	00:00:00.02
	Dimensions Requested	2
	Cells Available	174762

Case Processing Summary

	Cases					
	Valid		Missing		Total	
	N	Percent	N	Percent	N	Percent
Study Group * 30-Day Hospital Readmission	700	100.0%	0	0.0%	700	100.0%

Study Group * 30-Day Hospital Readmission Crosstabulation

			30-Day Hospital Readmission		Total
			0	1	
Study Group	1	Count	263	71	334
		% within Study Group	78.7%	21.3%	100.0%
		% within 30-Day Hospital Readmission	43.8%	71.7%	47.7%
		% of Total	37.6%	10.1%	47.7%
	2	Count	338	28	366
		% within Study Group	92.3%	7.7%	100.0%
		% within 30-Day Hospital Readmission	56.2%	28.3%	52.3%
		% of Total	48.3%	4.0%	52.3%
Total		Count	601	99	700
		% within Study Group	85.9%	14.1%	100.0%
		% within 30-Day Hospital Readmission	100.0%	100.0%	100.0%
		% of Total	85.9%	14.1%	100.0%

Chi-Square Tests

	Value	df	Asymp. Sig. (2-sided)	Exact Sig. (2-sided)	Exact Sig. (1-sided)
Pearson Chi-Square	26.629[a]	1	.000		
Continuity Correction[b]	25.520	1	.000		
Likelihood Ratio	27.234	1	.000		
Fisher's Exact Test				.000	.000
Linear-by-Linear Association	26.591	1	.000		
N of Valid Cases	700				

a. 0 cells (0.0%) have expected count less than 5. The minimum expected count is 47.24.

b. Computed only for a 2x2 table

```
CROSSTABS
 /TABLES=group BY adverse_event
 /FORMAT=AVALUE TABLES
 /STATISTICS=CHISQ
 /CELLS=COUNT ROW COLUMN TOTAL.
```

Crosstabs

Notes

Output Created		30-AUG-2025 17:31:59
Comments		
Input	Data	C:\Users\zshah\Desktop\depart annual ealuation 2024-25\Dr.ilyas project\hospital medication\asp_study_700.csv
	Filter	<none>
	Weight	<none>
	Split File	<none>
	N of Rows in Working Data File	700
Missing Value Handling	Definition of Missing	User-defined missing values are treated as missing.
	Cases Used	Statistics for each table are based on all the cases with valid data in the specified range(s) for all variables in each table.
Syntax		CROSSTABS /TABLES=group BY adverse_event /FORMAT=AVALUE TABLES /STATISTICS=CHISQ /CELLS=COUNT ROW COLUMN TOTAL.
Resources	Processor Time	00:00:00.00
	Elapsed Time	00:00:00.00
	Dimensions Requested	2
	Cells Available	174762

Case Processing Summary

	Cases					
	Valid		Missing		Total	
	N	Percent	N	Percent	N	Percent
Study Group * Antibiotic-Related Adverse Events	700	100.0%	0	0.0%	700	100.0%

Study Group * Antibiotic-Related Adverse Events Crosstabulation

			Antibiotic-Related Adverse Events		
			0	1	Total
Study Group	1	Count	287	47	334
		% within Study Group	85.9%	14.1%	100.0%
		% within Antibiotic-Related Adverse Events	46.2%	59.5%	47.7%
		% of Total	41.0%	6.7%	47.7%
	2	Count	334	32	366
		% within Study Group	91.3%	8.7%	100.0%
		% within Antibiotic-Related Adverse Events	53.8%	40.5%	52.3%
		% of Total	47.7%	4.6%	52.3%
Total		Count	621	79	700
		% within Study Group	88.7%	11.3%	100.0%
		% within Antibiotic-Related Adverse Events	100.0%	100.0%	100.0%
		% of Total	88.7%	11.3%	100.0%

Chi-Square Tests

	Value	df	Asymp. Sig. (2-sided)	Exact Sig. (2-sided)	Exact Sig. (1-sided)
Pearson Chi-Square	4.953[a]	1	.026		
Continuity Correction[b]	4.435	1	.035		
Likelihood Ratio	4.963	1	.026		
Fisher's Exact Test				.031	.018
Linear-by-Linear Association	4.946	1	.026		
N of Valid Cases	700				

a. 0 cells (0.0%) have expected count less than 5. The minimum expected count is 37.69.

b. Computed only for a 2x2 table

```
LOGISTIC REGRESSION
 /DEPENDENT clinical_cure
 /METHOD=ENTER group age charlson_score psi_score
 /PRINT=CI(95)
 /CRITERIA=PIN(.05) POUT(.10) ITERATE(20) CUT(.5)
 /CASEWISE OUTLIER(2).
```

Logistic Regression

Notes

Output Created		30-AUG-2025 17:31:59
Comments		
Input	Data	C:<br>\Users\zshah\Desktop\depart annual ealuation 2024-25\Dr.ilyas project\hospital medication\asp_study_700.csv
	Filter	<none>
	Weight	<none>
	Split File	<none>
	N of Rows in Working Data File	700
Syntax		LOGISTIC REGRESSION<br>/DEPENDENT clinical_cure<br>/METHOD=ENTER group age charlson_score psi_score<br>/PRINT=CI(95)<br>/CRITERIA=PIN(.05) POUT(.10) ITERATE(20) CUT(.5)<br>/CASEWISE OUTLIER...
Resources	Processor Time	00:00:00.00
	Elapsed Time	00:00:00.00

```
T-TEST GROUPS=group(1 2)
 /VARIABLES=age charlson_score psi_score
 /STATISTICS DESCRIPTIVES
 /CRITERIA=CI(.95).
```

T-Test

Notes

Output Created		30-AUG-2025 17:31:59
Comments		
Input	Data	C: \Users\zshah\Desktop\depart annual ealuation 2024-25\Dr.ilyas project\hospital medication\asp_study_700.csv
	Filter	<none>
	Weight	<none>
	Split File	<none>
	N of Rows in Working Data File	700
Missing Value Handling	Definition of Missing	User defined missing values are treated as missing.
	Cases Used	Statistics for each analysis are based on the cases with no missing or out-of-range data for any variable in the analysis.
Syntax		T-TEST GROUPS=group (1 2)<br>/VARIABLES=age charlson_score psi_score<br>/STATISTICS DESCRIPTIVES<br>/CRITERIA=CI(.95).
Resources	Processor Time	00:00:00.02
	Elapsed Time	00:00:00.03

Group Statistics

	Study Group	N	Mean	Std. Deviation	Std. Error Mean
Age (years)	1	334	64.85	12.144	.665
	2	366	65.46	11.798	.617
Charlson Comorbidity Index	1	334	4.44	2.842	.156
	2	366	4.51	2.980	.156
Pneumonia Severity Index	1	334	3.00	1.453	.080
	2	366	3.13	1.415	.074

Independent Samples Test

		Levene's Test for Equality of Variances		t-test for Equality of
		F	Sig.	t
Age (years)	Equal variances assumed	.667	.414	-.669
	Equal variances not assumed			-.668
Charlson Comorbidity Index	Equal variances assumed	2.117	.146	-.295
	Equal variances not assumed			-.296
Pneumonia Severity Index	Equal variances assumed	.004	.949	-1.262
	Equal variances not assumed			-1.261

Independent Samples Test

		t-test for Equality of Means		
		df	Sig. (2-tailed)	Mean Difference
Age (years)	Equal variances assumed	698	.504	-.606
	Equal variances not assumed	688.005	.504	-.606
Charlson Comorbidity Index	Equal variances assumed	698	.768	-.065
	Equal variances not assumed	696.630	.768	-.065
Pneumonia Severity Index	Equal variances assumed	698	.207	-.137
	Equal variances not assumed	688.363	.208	-.137

Independent Samples Test

		t-test for Equality of Means	
		Std. Error Difference	95% Confidence ...
			Lower
Age (years)	Equal variances assumed	.905	-2.383
	Equal variances not assumed	.907	-2.386
Charlson Comorbidity Index	Equal variances assumed	.221	-.498
	Equal variances not assumed	.220	-.497
Pneumonia Severity Index	Equal variances assumed	.108	-.350
	Equal variances not assumed	.109	-.350

Independent Samples Test

		t-test for Equality of ...
		95% Confidence ...
		Upper
Age (years)	Equal variances assumed	1.172
	Equal variances not assumed	1.174
Charlson Comorbidity Index	Equal variances assumed	.368
	Equal variances not assumed	.367
Pneumonia Severity Index	Equal variances assumed	.076
	Equal variances not assumed	.076

```
T-TEST GROUPS=group(1 2)
 /VARIABLES=LOS antibiotic_days
 /STATISTICS DESCRIPTIVES
 /ES DISPLAY(TRUE).
```

T-Test

Notes

Output Created		30-AUG-2025 17:31:59
Comments		
Input	Data	C:<br>\Users\zshah\Desktop\depart annual ealuation 2024-25\Dr.ilyas project\hospital medication\asp_study_700.csv
	Filter	<none>
	Weight	<none>
	Split File	<none>
	N of Rows in Working Data File	700
Missing Value Handling	Definition of Missing	User defined missing values are treated as missing.
	Cases Used	Statistics for each analysis are based on the cases with no missing or out-of-range data for any variable in the analysis.
Syntax		T-TEST GROUPS=group (1 2)<br>/VARIABLES=LOS antibiotic_days<br>/STATISTICS DESCRIPTIVES<br>/ES DISPLAY(TRUE).
Resources	Processor Time	00:00:00.00
	Elapsed Time	00:00:00.03

Group Statistics

	Study Group	N	Mean	Std. Deviation	Std. Error Mean
Length of Hospital Stay (days)	1	334	17.47	7.218	.395
	2	366	14.64	5.479	.286
Total Antibiotic Treatment Days	1	334	13.89	6.200	.339
	2	366	10.23	4.780	.250

Independent Samples Test

		Levene's Test for Equality of Variances		t-test for Equality of
		F	Sig.	t
Length of Hospital Stay (days)	Equal variances assumed	38.514	.000	5.862
	Equal variances not assumed			5.790
Total Antibiotic Treatment Days	Equal variances assumed	26.538	.000	8.788
	Equal variances not assumed			8.686

Independent Samples Test

		t-test for Equality of Means		
		df	Sig. (2-tailed)	Mean Difference
Length of Hospital Stay (days)	Equal variances assumed	698	.000	2.825
	Equal variances not assumed	619.115	.000	2.825
Total Antibiotic Treatment Days	Equal variances assumed	698	.000	3.660
	Equal variances not assumed	624.551	.000	3.660

Independent Samples Test

		t-test for Equality of Means	
		Std. Error Difference	95% Confidence ...
			Lower
Length of Hospital Stay (days)	Equal variances assumed	.482	1.879
	Equal variances not assumed	.488	1.867
Total Antibiotic Treatment Days	Equal variances assumed	.416	2.842
	Equal variances not assumed	.421	2.832

Independent Samples Test

		t-test for Equality of ...
		95% Confidence ...
		Upper
Length of Hospital Stay (days)	Equal variances assumed	3.771
	Equal variances not assumed	3.783
Total Antibiotic Treatment Days	Equal variances assumed	4.477
	Equal variances not assumed	4.487

```
CORRELATIONS
  /VARIABLES=age charlson_score psi_score LOS antibiotic_days
  /PRINT=TWOTAIL NOSIG
  /STATISTICS DESCRIPTIVES.
```

Correlations

Notes

Output Created		30-AUG-2025 17:31:59
Comments		
Input	Data	C: \Users\zshah\Desktop\depart annual ealuation 2024-25\Dr.ilyas project\hospital medication\asp_study_700.csv
	Filter	<none>
	Weight	<none>
	Split File	<none>
	N of Rows in Working Data File	700
Missing Value Handling	Definition of Missing	User-defined missing values are treated as missing.
	Cases Used	Statistics for each pair of variables are based on all the cases with valid data for that pair.

Notes

Syntax		CORRELATIONS<br>/VARIABLES=age charlson_score psi_score LOS antibiotic_days<br>/PRINT=TWOTAIL NOSIG<br>/STATISTICS ...
Resources	Processor Time	00:00:00.00
	Elapsed Time	00:00:00.00

Descriptive Statistics

	Mean	Std. Deviation	N
Age (years)	65.17	11.960	700
Charlson Comorbidity Index	4.48	2.913	700
Pneumonia Severity Index	3.07	1.434	700
Length of Hospital Stay (days)	15.99	6.518	700
Total Antibiotic Treatment Days	11.98	5.796	700

Correlations

		Age (years)	Charlson Comorbidity Index	Pneumonia Severity Index
Age (years)	Pearson Correlation	1	.036	.009
	Sig. (2-tailed)		.336	.807
	N	700	700	700
Charlson Comorbidity Index	Pearson Correlation	.036	1	.027
	Sig. (2-tailed)	.336		.474
	N	700	700	700
Pneumonia Severity Index	Pearson Correlation	.009	.027	1
	Sig. (2-tailed)	.807	.474	
	N	700	700	700
Length of Hospital Stay (days)	Pearson Correlation	.016	-.048	-.071
	Sig. (2-tailed)	.681	.208	.061
	N	700	700	700
Total Antibiotic Treatment Days	Pearson Correlation	-.031	.006	.057
	Sig. (2-tailed)	.412	.867	.129
	N	700	700	700

Correlations

		Length of Hospital Stay (days)	Total Antibiotic Treatment Days
Age (years)	Pearson Correlation	.016	-.031
	Sig. (2-tailed)	.681	.412
	N	700	700
Charlson Comorbidity Index	Pearson Correlation	-.048	.006
	Sig. (2-tailed)	.208	.867
	N	700	700
Pneumonia Severity Index	Pearson Correlation	-.071	.057
	Sig. (2-tailed)	.061	.129
	N	700	700
Length of Hospital Stay (days)	Pearson Correlation	1	.089*
	Sig. (2-tailed)		.019
	N	700	700
Total Antibiotic Treatment Days	Pearson Correlation	.089*	1
	Sig. (2-tailed)	.019	
	N	700	700

*. Correlation is significant at the 0.05 level (2-tailed).

```
MEANS TABLES=age charlson_score psi_score LOS antibiotic_days BY group
  /CELLS=MEAN STDDEV COUNT.
```

Means

Notes

Output Created		30-AUG-2025 17:31:59
Comments		
Input	Data	C:\Users\zshah\Desktop\depart annual ealuation 2024-25\Dr.ilyas project\hospital medication\asp_study_700.csv
	Filter	<none>
	Weight	<none>
	Split File	<none>
	N of Rows in Working Data File	700
Missing Value Handling	Definition of Missing	For each dependent variable in a table, user-defined missing values for the dependent and all grouping variables are treated as missing.
	Cases Used	Cases used for each table have no missing values in any independent variable, and not all dependent variables have missing values.
Syntax		MEANS TABLES=age charlson_score psi_score LOS antibiotic_days BY group /CELLS=MEAN STDDEV COUNT.
Resources	Processor Time	00:00:00.00
	Elapsed Time	00:00:00.00

Case Processing Summary

	Cases					
	Included		Excluded		Total	
	N	Percent	N	Percent	N	Percent
Age (years) * Study Group	700	100.0%	0	0.0%	700	100.0%
Charlson Comorbidity Index * Study Group	700	100.0%	0	0.0%	700	100.0%
Pneumonia Severity Index * Study Group	700	100.0%	0	0.0%	700	100.0%
Length of Hospital Stay (days) * Study Group	700	100.0%	0	0.0%	700	100.0%
Total Antibiotic Treatment Days * Study Group	700	100.0%	0	0.0%	700	100.0%

Report

Study Group		Age (years)	Charlson Comorbidity Index	Pneumonia Severity Index	Length of Hospital Stay (days)
1	Mean	64.85	4.44	3.00	17.47
	Std. Deviation	12.144	2.842	1.453	7.218
	N	334	334	334	334
2	Mean	65.46	4.51	3.13	14.64
	Std. Deviation	11.798	2.980	1.415	5.479
	N	366	366	366	366
Total	Mean	65.17	4.48	3.07	15.99
	Std. Deviation	11.960	2.913	1.434	6.518
	N	700	700	700	700

Report

Study Group		Total Antibiotic Treatment Days
1	Mean	13.89
	Std. Deviation	6.200
	N	334
2	Mean	10.23
	Std. Deviation	4.780
	N	366
Total	Mean	11.98
	Std. Deviation	5.796
	N	700

```
FREQUENCIES VARIABLES=ALL
/FORMAT=NOTABLE
/STATISTICS=VALID MISSING.
```

Frequencies

Notes

Output Created		30-AUG-2025 17:31:59
Comments		
Input	Data	C: \Users\zshah\Desktop\depart annual ealuation 2024-25\Dr.ilyas project\hospital medication\asp_study_700.csv
	Filter	<none>
	Weight	<none>
	Split File	<none>
	N of Rows in Working Data File	700
Missing Value Handling	Definition of Missing	User-defined missing values are treated as missing.
	Cases Used	Statistics are based on all cases with valid data.
Syntax		FREQUENCIES VARIABLES=ALL /FORMAT=NOTABLE /STATISTICS=VALID MISSING.
Resources	Processor Time	00:00:00.00
	Elapsed Time	00:00:00.00

Warnings

The STATISTICS subcommand has encountered a bad option keyword. Found: VALID
The STATISTICS subcommand has encountered a bad option keyword. Found: MISSING

Statistics

		Patient ID	Study Group	Age (years)	Charlson Comorbidity Index	Pneumonia Severity Index
N	Valid	700	700	700	700	700
	Missing	0	0	0	0	0
Mean		350.50	1.52	65.17	4.48	3.07
Std. Deviation		202.217	.500	11.960	2.913	1.434
Minimum		1	1	45	0	1
Maximum		700	2	85	9	5

Statistics

		Appropriate Antibiotic Prescribing (Day 3)	Clinical Cure (Day 14)	Length of Hospital Stay (days)	Total Antibiotic Treatment Days	30-Day Hospital Readmission
N	Valid	700	700	700	700	700
	Missing	0	0	0	0	0
Mean		.70	.80	15.99	11.98	.14
Std. Deviation		.460	.403	6.518	5.796	.349
Minimum		0	0	4	2	0
Maximum		1	1	29	24	1

Statistics

		Antibiotic-Related Adverse Events
N	Valid	700
	Missing	0
Mean		.11
Std. Deviation		.317
Minimum		0
Maximum		1

```
DESCRIPTIVES VARIABLES=ALL
/STATISTICS=MEAN STDDEV MIN MAX RANGE.
```

Descriptives

Notes

Output Created		30-AUG-2025 17:31:59
Comments		
Input	Data	C: \Users\zshah\Desktop\depart annual ealuation 2024-25\Dr.ilyas project\hospital medication\asp_study_700.csv
	Filter	<none>
	Weight	<none>
	Split File	<none>
	N of Rows in Working Data File	700
Missing Value Handling	Definition of Missing	User defined missing values are treated as missing.
	Cases Used	All non-missing data are used.
Syntax		DESCRIPTIVES VARIABLES=ALL /STATISTICS=MEAN STDDEV MIN MAX RANGE.
Resources	Processor Time	00:00:00.00
	Elapsed Time	00:00:00.00

Descriptive Statistics

	N	Range	Minimum	Maximum	Mean	Std. Deviation
Patient ID	700	699	1	700	350.50	202.217
Study Group	700	1	1	2	1.52	.500
Age (years)	700	40	45	85	65.17	11.960
Charlson Comorbidity Index	700	9	0	9	4.48	2.913
Pneumonia Severity Index	700	4	1	5	3.07	1.434
Appropriate Antibiotic Prescribing (Day 3)	700	1	0	1	.70	.460
Clinical Cure (Day 14)	700	1	0	1	.80	.403
Length of Hospital Stay (days)	700	25	4	29	15.99	6.518
Total Antibiotic Treatment Days	700	22	2	24	11.98	5.796
30-Day Hospital Readmission	700	1	0	1	.14	.349
Antibiotic-Related Adverse Events	700	1	0	1	.11	.317
Valid N (listwise)	700					

```
LOGISTIC REGRESSION VARIABLES appropriate
  /METHOD=ENTER group
  /PRINT=CI(95)
  /CRITERIA=PIN(.05) POUT(.10) ITERATE(20) CUT(.5).
```

Logistic Regression

Notes

Output Created		30-AUG-2025 17:32:00
Comments		
Input	Data	C: \Users\zshah\Desktop\depart annual ealuation 2024-25\Dr.ilyas project\hospital medication\asp_study_700.csv
	Filter	<none>
	Weight	<none>
	Split File	<none>
	N of Rows in Working Data File	700
Missing Value Handling	Definition of Missing	User-defined missing values are treated as missing
Syntax		LOGISTIC REGRESSION VARIABLES appropriate /METHOD=ENTER group /PRINT=CI(95) /CRITERIA=PIN(.05) POUT(.10) ITERATE(20) CUT(.5).
Resources	Processor Time	00:00:00.03
	Elapsed Time	00:00:00.02

Case Processing Summary

Unweighted Cases[a]		N	Percent
Selected Cases	Included in Analysis	700	100.0
	Missing Cases	0	.0
	Total	700	100.0
Unselected Cases		0	.0
Total		700	100.0

a. If weight is in effect, see classification table for the total number of cases.

Dependent Variable Encoding

Original Value	Internal Value
0	0
1	1

Block 0: Beginning Block

Classification Table[a,b]

Observed			Predicted: Appropriate Antibiotic Prescribing (Day 3) 0	1	Percentage Correct
Step 0	Appropriate Antibiotic Prescribing (Day 3)	0	0	213	.0
		1	0	487	100.0
	Overall Percentage				69.6

a. Constant is included in the model.

b. The cut value is .500

Variables in the Equation

		B	S.E.	Wald	df	Sig.	Exp(B)
Step 0	Constant	.827	.082	101.343	1	.000	2.286

Variables not in the Equation

			Score	df	Sig.
Step 0	Variables	group	23.330	1	.000
	Overall Statistics		23.330	1	.000

Block 1: Method = Enter

Omnibus Tests of Model Coefficients

		Chi-square	df	Sig.
Step 1	Step	23.446	1	.000
	Block	23.446	1	.000
	Model	23.446	1	.000

Model Summary

Step	-2 Log likelihood	Cox & Snell R Square	Nagelkerke R Square
1	836.787[a]	.033	.047

a. Estimation terminated at iteration number 4 because parameter estimates changed by less than .001.

Classification Table[a]

Observed			Predicted: Appropriate Antibiotic Prescribing (Day 3) 0	1	Percentage Correct
Step 1	Appropriate Antibiotic Prescribing (Day 3)	0	0	213	.0
		1	0	487	100.0
	Overall Percentage				69.6

a. The cut value is .500

Variables in the Equation

		B	S.E.	Wald	df	Sig.	Exp(B)	95% C.I... Lower
Step 1[a]	group	.804	.168	22.875	1	.000	2.235	1.607
	Constant	-.366	.257	2.034	1	.154	.693	

Variables in the Equation

		95% C.I.... Upper
Step 1[a]	group	3.108
	Constant	

a. Variable(s) entered on step 1: group.

```
LOGISTIC REGRESSION VARIABLES clinical_cure
 /METHOD=ENTER group
 /PRINT=CI(95)
 /CRITERIA=PIN(.05) POUT(.10) ITERATE(20) CUT(.5).
```

Logistic Regression

Notes

Output Created		30-AUG-2025 17:32:00
Comments		
Input	Data	C:<br>\Users\zshah\Desktop\depart annual ealuation 2024-25\Dr.ilyas project\hospital medication\asp_study_700.csv
	Filter	<none>
	Weight	<none>
	Split File	<none>
	N of Rows in Working Data File	700
Missing Value Handling	Definition of Missing	User-defined missing values are treated as missing
Syntax		LOGISTIC REGRESSION VARIABLES clinical_cure<br>/METHOD=ENTER group<br>/PRINT=CI(95)<br>/CRITERIA=PIN(.05) POUT(.10) ITERATE(20) CUT(.5).
Resources	Processor Time	00:00:00.02
	Elapsed Time	00:00:00.02

Case Processing Summary

Unweighted Cases[a]		N	Percent
Selected Cases	Included in Analysis	700	100.0
	Missing Cases	0	.0
	Total	700	100.0
Unselected Cases		0	.0
Total		700	100.0

a. If weight is in effect, see classification table for the total number of cases.

Dependent Variable Encoding

Original Value	Internal Value
0	0
1	1

Block 0: Beginning Block

Classification Table[a,b]

Observed			Predicted: Clinical Cure (Day 14) 0	1	Percentage Correct
Step 0	Clinical Cure (Day 14)	0	0	143	.0
		1	0	557	100.0
	Overall Percentage				79.6

a. Constant is included in the model.

b. The cut value is .500

Variables in the Equation

		B	S.E.	Wald	df	Sig.	Exp(B)
Step 0	Constant	1.360	.094	210.374	1	.000	3.895

Variables not in the Equation

			Score	df	Sig.
Step 0	Variables	group	11.122	1	.001
	Overall Statistics		11.122	1	.001

Block 1: Method = Enter

Omnibus Tests of Model Coefficients

		Chi-square	df	Sig.
Step 1	Step	11.153	1	.001
	Block	11.153	1	.001
	Model	11.153	1	.001

Model Summary

Step	-2 Log likelihood	Cox & Snell R Square	Nagelkerke R Square
1	697.649[a]	.016	.025

a. Estimation terminated at iteration number 4 because parameter estimates changed by less than .001.

Classification Table[a]

Observed			Predicted: Clinical Cure (Day 14) 0	1	Percentage Correct
Step 1	Clinical Cure (Day 14)	0	0	143	.0
		1	0	557	100.0
	Overall Percentage				79.6

a. The cut value is .500

Variables in the Equation

		B	S.E.	Wald	df	Sig.	Exp(B)	95% C.I....
								Lower
Step 1[a]	group	.631	.191	10.934	1	.001	1.880	1.293
	Constant	.428	.289	2.195	1	.138	1.534	

Variables in the Equation

		95% C.I....
		Upper
Step 1[a]	group	2.733
	Constant	

a. Variable(s) entered on step 1: group.

```
LOGISTIC REGRESSION VARIABLES clinical_cure
 /METHOD=ENTER group age charlson_score psi_score
 /PRINT=CI(95)
 /CRITERIA=PIN(.05) POUT(.10) ITERATE(20) CUT(.5).
```

Logistic Regression

Notes

Output Created		30-AUG-2025 17:32:00
Comments		
Input	Data	C:\Users\zshah\Desktop\depart annual eualuation 2024-25\Dr.ilyas project\hospital medication\asp_study_700.csv
	Filter	<none>
	Weight	<none>
	Split File	<none>
	N of Rows in Working Data File	700
Missing Value Handling	Definition of Missing	User-defined missing values are treated as missing
Syntax		LOGISTIC REGRESSION VARIABLES clinical_cure /METHOD=ENTER group age charlson_score psi_score /PRINT=CI(95) /CRITERIA=PIN(.05) POUT(.10) ITERATE(20) CUT(.5).

Notes

Resources	Processor Time	00:00:00.02
	Elapsed Time	00:00:00.02

Case Processing Summary

Unweighted Cases[a]		N	Percent
Selected Cases	Included in Analysis	700	100.0
	Missing Cases	0	.0
	Total	700	100.0
Unselected Cases		0	.0
Total		700	100.0

a. If weight is in effect, see classification table for the total number of cases.

Dependent Variable Encoding

Original Value	Internal Value
0	0
1	1

Block 0: Beginning Block

Classification Table[a,b]

	Observed		Predicted		
			Clinical Cure (Day 14)		Percentage Correct
			0	1	
Step 0	Clinical Cure (Day 14)	0	0	143	.0
		1	0	557	100.0
	Overall Percentage				79.6

a. Constant is included in the model.

b. The cut value is .500

Variables in the Equation

		B	S.E.	Wald	df	Sig.	Exp(B)
Step 0	Constant	1.360	.094	210.374	1	.000	3.895

Variables not in the Equation

			Score	df	Sig.
Step 0	Variables	group	11.122	1	.001
		age	3.673	1	.055
		charlson_score	.538	1	.463
		psi_score	.075	1	.784
	Overall Statistics		15.364	4	.004

Block 1: Method = Enter

Omnibus Tests of Model Coefficients

		Chi-square	df	Sig.
Step 1	Step	15.445	4	.004
	Block	15.445	4	.004
	Model	15.445	4	.004

Model Summary

Step	-2 Log likelihood	Cox & Snell R Square	Nagelkerke R Square
1	693.356[a]	.022	.034

a. Estimation terminated at iteration number 4 because parameter estimates changed by less than .001.

Classification Table[a]

Observed			Predicted		
			Clinical Cure (Day 14)		Percentage Correct
			0	1	
Step 1	Clinical Cure (Day 14)	0	0	143	.0
		1	0	557	100.0
	Overall Percentage				79.6

a. The cut value is .500

Variables in the Equation

		B	S.E.	Wald	df	Sig.	Exp(B)
Step 1[a]	group	.631	.192	10.830	1	.001	1.879
	age	.015	.008	3.489	1	.062	1.015
	charlson_score	-.028	.033	.709	1	.400	.973
	psi_score	-.027	.066	.167	1	.682	.973
	Constant	-.321	.624	.265	1	.607	.725

Variables in the Equation

		95% C.I.for EXP(B)	
		Lower	Upper
Step 1[a]	group	1.291	2.737
	age	.999	1.031
	charlson_score	.912	1.037
	psi_score	.855	1.108
	Constant		

a. Variable(s) entered on step 1: group, age, charlson_score, psi_score.
