# Supplementary material for: Impact of clinical pharmacist-led antimicrobial stewardship on antibiotic appropriateness, clinical outcomes, and antimicrobial consumption in hospital-acquired and ventilator-associated pneumonia: a randomized controlled trial
Source: Front Public Health. 2026 Apr 7;14:1700328. doi: 10.3389/fpubh.2026.1700328 (PMC13095787; doi:10.3389/fpubh.2026.1700328)
Supplement: Supplementary file 2 [file Table_1.DOCX]

**Supplementary Table S1.** Operational Criteria for Antimicrobial Appropriateness Assessment

| Domain | Criteria | Scoring |
| --- | --- | --- |
| Drug Selection | Spectrum covers cultured organisms OR appropriate empirical coverage if cultures pending/negative | Appropriate/Inappropriate |
| Dosing | Dose adjusted for renal/hepatic function, body weight, severity of infection per PK/PD principles | Appropriate/Inappropriate |
| Route | IV to PO switch criteria met where applicable; IV continuation justified | Appropriate/Inappropriate |
| Combination Therapy | Justified for severe infection, MDR risk, or synergy; unnecessary duplication avoided | Appropriate/Inappropriate |
| De-escalation | Narrow-spectrum agent used when susceptibility allows | Appropriate/Inappropriate |
| Duration Plan | Documented duration aligned with guidelines (7-8 days HAP, 7-14 days VAP) | Appropriate/Inappropriate |

Overall Appropriateness = All domains appropriate
